# Genetic Evidence for Roles of Yeast Mitotic Cyclins at Single-Stranded Gaps Created by DNA Replication

**DOI:** 10.1534/g3.117.300537

**Published:** 2017-12-26

**Authors:** Laurence Signon

**Affiliations:** Institute for Integrative Biology of the Cell (I2BC), CEA, CNRS, University of Paris-Sud, Université Paris-Saclay, 91198 Gif-sur-Yvette, France

**Keywords:** mitotic cyclin, replication, hydroxyurea, stalled forks, SGS1 helicase, EXO1 nuclease

## Abstract

Paused or stalled replication forks are major threats to genome integrity; unraveling the complex pathways that contribute to fork stability and restart is crucial. Experimentally, fork stalling is induced by growing the cells in presence of hydroxyurea (HU), which depletes the pool of deoxynucleotide triphosphates (dNTPs) and slows down replication progression in yeast. Here, I report an epistasis analysis, based on sensitivity to HU, between *CLB2*, the principal mitotic cyclin gene in *Saccharomyces cerevisiae*, and genes involved in fork stability and recombination. *clb2*Δ cells are not sensitive to HU, but the strong synergistic effect of *clb2*Δ with most genes tested indicates, unexpectedly, that *CLB2* has an important role in DNA replication, in the stability and restart of stalled forks, and in pathways dependent on and independent of homologous recombination. Results indicate that *CLB2* functions in parallel with the *SGS1* helicase and *EXO1* exonuclease to allow proper Rad51 recombination, but also regulates a combined Sgs1–Exo1 activity in a pathway dependent on Mec1 and Rad53 checkpoint protein kinases. The data argue that Mec1 regulates Clb2 to prevent a deleterious Sgs1–Exo1 activity at paused or stalled forks, whereas Rad53 checkpoint activation regulates Clb2 to allow a necessary Sgs1–Exo1 activity at stalled or collapsed forks. Altogether, this study indicates that Clb2 regulates the activity of numerous nucleases at single-stranded gaps created by DNA replication. A model is proposed for the function and regulation of Clb2 at stalled forks. These data provide new perspectives on the role of mitotic cyclins at the end of S phase.

During replication, fork progression frequently slows down or stalls owing to the presence of replication barriers, such as replication slow zones (RSZs), secondary DNA structures, protein–DNA complexes, and gene transcription (Durkin and Glover 2007; Zeman and Cimprich 2014). Correct and processive fork progression is dependent on an adequate pool of deoxynucleotide triphosphates (dNTPs). Initiation of DNA replication, without a sufficient nucleotide pool, results in slowing down and stalling of replication forks and increasing genetic instability, as observed in early-stage cancer upon oncogene expression (Bester *et al.* 2011). Replication stress can be induced experimentally by a ribonucleotide reductase inhibitor, hydroxyurea (HU), which results in the depletion of dNTPs, thereby causing a large decrease in the replication fork rate and increased amounts of single-stranded DNA (ssDNA) at replication forks (Sogo *et al.* 2002; Tercero *et al.* 2003; Feng *et al.* 2006). Polymerase stalling results in uncoupling of DNA polymerase and the replicative helicase, which generates ssDNA (Byun *et al.* 2005; Zeman and Cimprich 2014). Accumulation of ssDNA at stalled replication forks triggers the replication checkpoint, which permits fork stabilization and delays cell cycle progression until S phase is complete (Zeman and Cimprich 2014; Weinert *et al.* 1994; Desany *et al.* 1998; Lopes *et al.* 2001). The two essential yeast protein kinases Mec1 and Rad53 (ATR and CHK2 in mammals) play essential parts in these processes (Weinert *et al.* 1994; Desany *et al.* 1998; Lopes *et al.* 2001; Friedel *et al.* 2009). The sensor kinase Mec1 is recruited by replication protein A, which binds to ssDNA at stalled forks (Friedel *et al.* 2009; Zeman and Cimprich 2014). Once recruited, Mec1 phosphorylates the effector kinase Rad53 (Sun *et al.* 1996; Sanchez *et al.* 1996; Friedel *et al.* 2009). Mec1 and Rad53 then regulate numerous DNA replication or repair proteins to preserve both the structural integrity of replication intermediates and the proficiency for DNA synthesis of stalled forks (Friedel *et al.* 2009; Zeman and Cimprich 2014; Cortez 2015). *mec1* and *rad53* mutants are extremely sensitive to HU and die owing to irreversible fork collapse. Mec1 and Rad53 prevent nucleolytic degradation at stalled forks by regulating nucleases and DNA-processing enzymes, including the exonuclease Exo1, responsible for fork collapse and double strand break (DSB) formation (Cotta-Ramusino *et al.* 2005; Kai *et al.* 2005; Trenz *et al.* 2006; Froget *et al.* 2008; Segurado and Diffley 2008; Friedel *et al.* 2009, Zeman and Cimprich 2014). Exo1 is associated with forks and is responsible for the ssDNA accumulation and aberrant fork structure found in *rad53*Δ cells in response to methyl methanesulfonate (MMS) and HU (Cotta-Ramusino *et al.* 2005; Segurado and Diffley 2008). However, although deletion of *EXO1* rescues *rad53*Δ cell lethality in response to MMS, it does not have the same effect in HU-induced lethality, indicating that forks stalled by HU are processed differently, in part, from forks blocked by MMS (Segurado and Diffley 2008). In addition, deletion of *EXO1* does not rescue *mec1*Δ cell lethality in response to MMS or HU, suggesting that Mec1 and Rad53 have separate functions at the fork (Segurado and Diffley 2008). Exo1 is hyper-phosphorylated upon HU treatment in a Mec1-dependent manner (Engels *et al.* 2011). Exo1 possesses 5′ to 3′ exonuclease activity on double-stranded DNA (dsDNA) as well as a flap-endonuclease activity. Exo1 has been implicated in several DNA repair pathways including mismatch repair, postreplication repair, mitotic recombination, and DSB repair (Szankasi and Smith 1995; Tsubouchi and Ogawa 2000; Mimitou and Symington 2008; Zhu *et al.* 2008).

The RecQ DNA helicase family plays critical parts during replication in preserving the integrity of stalled replication forks, and its loss has been associated with human diseases (Croteau *et al.* 2014). Sgs1 is a yeast member of this family. The enzymatic activities of Sgs1 that have been characterized *in vitro* and *in vivo* include the annealing of complementary strands of DNA, branch migration, regression of replication forks, and resolution of Holliday junctions that form at a collapsed replication fork or at recombinant structures (Kaliraman *et al.* 2001; Ralf *et al.* 2006; Gravel *et al.* 2008; Croteau *et al.* 2014). Sgs1 is also involved in long-range DNA end resection at DSBs in association with Dna2 nuclease, and functions in parallel with Exo1 to promote homologous recombination (HR) (Gravel *et al.* 2008; Mimitou and Symington 2008; Zhu *et al.* 2008). By contrast, at stalled forks and telomeres, Sgs1 prevents the accumulation of ssDNA and HR (Fabre *et al.* 2002; Ouyang *et al.* 2013; Hardy *et al.* 2014). Thus, Sgs1 helicase is central for both the stabilization and recovery of stalled replication forks.

In wild-type (WT) cells, after a short replication block, most forks resume progression. Prolonged stalling leads to fork inactivation and alternative pathways of fork restart, such as new origin firing (Ge *et al.* 2007) and template switching by HR-dependent mechanisms (Figure 1) (Saintigny *et al.* 2001; Lambert *et al.* 2010; Petermann *et al.* 2010; Carr and Lambert 2013). It has been suggested that fork reversal could be a transient physiological intermediate that accounts for fork protection and restart of stalled forks (Atkinson and McGlynn 2009; Carr and Lambert 2013; Zeman and Cimprich 2014). Reversed forks can be restarted by either exonucleolytic degradation or HR mechanisms (Figure 1). Failure to restart a fork by these mechanisms can induce one-ended DSBs, which can be repaired by HR mechanisms. In mammals, DSB formation increases with the duration of HU exposure (Saintigny *et al.* 2001; Petermann *et al.* 2010). By contrast, in budding yeast, HU-treated WT cells exhibit normal replication forks that sustain very slow DNA synthesis (Sogo *et al.* 2002; Tercero *et al.* 2003; Feng *et al.* 2006; Alvino *et al.* 2007), and a high level of DSBs is observed after HU removal, during recovery (Alvino *et al.* 2007; Hoffman *et al.* 2015).

**Figure 1:**
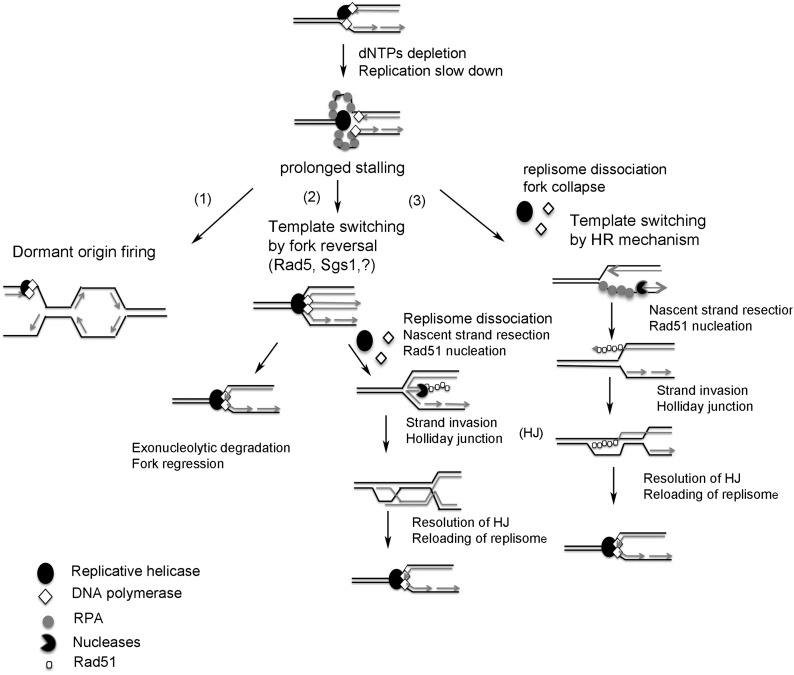
Models of replication restart of stalled forks. Depletion of dNTPs slows down replication fork processivity, and uncoupling between the helicase and polymerases leads to accumulation of ssDNA. Prolonged stalling leads to fork inactivation and an alternate pathway of fork restart, such as new origin firing (1), fork reversal (2), or template switching by HR mechanisms (3). (2) Template switching by fork reversal (*i.e.*, the fork moving backward with nascent strand being annealed together to form a four way junction) can occur by Rad5 or Sgs1 or by a yet-unknown pathway (?) and might help to protect the nascent strand from extensive resection. The resolution of fork reversal can occur either by exonucleolytic degradation or through the HR pathway. The molecular structure of stalled and collapsed forks remains uncertain, but replisome dissociation has been associated with fork collapse. (3) The dissociation of replisome allows nascent strand resection that might help the fork to regress (fork moving backward without nascent strand being annealed). Rad51 then nucleates on the exposed ssDNA and promotes HR with the parental duplex.

In eukaryotic cells, cell cycle progression is driven by the cyclin-dependent kinases (Cdks). Cdks interact with different cyclins throughout the cell cycle. Cyclins are essential activator of Cdks and are involved in the recruitment and selection of substrates. In budding yeast, six B-type cyclins (Clbs) associate with a single Cdk, Cdk1 (Cdc28), to drive S and M phase progression (Bloom and Cross 2007). Significant overlaps exist between the substrates that are phosphorylated by the various Clb–Cdk1 complexes, because overexpression of a single Clb [*e.g.*, Clb2 (Haase and Reed 1999) or Clb6 (Schwob *et al.* 1994)] can rescue the lethality of a *clb1*,*2*,*3*,*4*,*5*,*6*Δ mutant. Mitotic cyclins Clb1, Clb2, Clb3, and Clb4 drive mitosis initiation and progression. They are involved in cell morphogenesis (Howell and Lew 2012) and the regulation of microtubule dynamics during metaphase and anaphase (Surana *et al.* 1991; Fitch *et al.* 1992; Signon 2011), the inhibition of prereplication complex formation (Detweiler and Li 1998), and the regulation of DNA polymerase α primase association with chromatin (Desdouets *et al.* 1998). Recently, numerous reports have involved mitotic-Cdk1 in DNA repair and, more specifically, in the repair of DSBs (Caspari *et al.* 2002;). Mitotic-Cdk1 has been shown to control initial resection by regulating Sae2 nuclease and extensive resection by regulating Dna2 nuclease (Ira *et al.* 2004; Huertas *et al.* 2008; Chen *et al.* 2011); and to be involved in, the resolution of late recombinant structures by regulating Mus81 resolvase via phosphorylation of the noncatalytic Mms4 subunit (Matos *et al.* 2011; Gallo-Fernández *et al.* 2012). Finally, one study has implicated Clb2–Cdk1 in the *mcm5-bob1* bypass of Cdc7p/Dbf4 and suggested a specific role for Clb2–Cdk1 during replication (Sclafani *et al.* 2002). We also recently published a broad epistasis analysis between *CLB2* and genes involved in DNA repair, recombination, and signal transduction, based on sensitivity to the DNA-damaging agent MMS, which suggests that *CLB2* is involved at forks blocked by MMS and regulates, among other pathways, Sgs1 helicase and Exo1 nuclease activity (Signon and Simon 2014). In addition, that study indicated that Sgs1 is an important regulator of Exo1 activity and suggested that Sgs1 and Exo1 might form a complex. The sensitivity to replication inhibitor HU was assessed in parallel with the sensitivity to MMS (Signon and Simon 2014). Cells were plated on both MMS and HU plates. Contrary to MMS, which induces DNA damage, DNA DSBs, and G2/M checkpoint activation, chronic HU exposure slows down global replication progression in budding yeast and induces only the replication checkpoint (Sogo *et al.* 2002; Tercero *et al.* 2003; Feng *et al.* 2006; Alvino *et al.* 2007).

Here, I report the genetic interactions between *CLB2* and these genes (Signon and Simon 2014) in response to chronic HU exposure. This study indicates, unexpectedly, that Clb2 and mitotic cyclins have important roles in the process of replication, and that mitotic cyclins are involved in numerous pathways that contribute to the stability and restart of stalled forks. These data converge on the idea that Clb2 regulates nucleases activity at single-stranded gaps created by replication, including the activity of a complex formed by Sgs1 and Exo1. Most interestingly, this study reveals novel aspects of Sgs1–Exo1 regulation by Clb2 that depend on Mec1 and Rad53 checkpoint proteins. A model for the roles of Clb2 at stalled forks at the end of S phase is proposed.

## Materials and Methods

### Media and yeast strains

Standard procedures were used for mating, sporulation, tetrad dissection (Sherman *et al.* 1983), and yeast transformation (Ito *et al.* 1983).

Yeast strains used in this study are listed in Supplemental Material, Table S1 in File S1, are isogenic to BF264-J15DU (*MATa leu2 ura3 trp1 his2 ade1*), and are described in Signon and Simon (2014). The *RAD52*::*TRP* disruption was obtained using the vectors pMS21 cut with *Bam*^1^H (kindly provided by David Schild). The *dna2-1* strain was obtained by sporulation of a diploid YLS37x ORD5350-5B (kindly gifted by Alain Nicolas), backcrossed five times with WT YLS37. The *dna2-1* spores were identified by their thermo-sensitivity at 30°. For the quintuple mutants *mec1*Δ*sml1*Δ*exo1*Δ*sgs1*Δ*clb2*Δ, *rad53*Δ*sml1*Δ*exo1*Δ*sgs1*Δ*clb2*Δ, and *rad53K227Asml1*Δ*exo1*Δ *sgs1*Δ*clb2*Δ, to verify that mutant spores carry both the *CLB2*::*TRP1* and the *SML1*::*TRP1* markers, they were backcrossed with the isogenic WT type strain or alternative *HIS+* strain. Diploids were then sporulated and a few tetrads were dissected and analyzed. The presence of tetrads showing a segregation (3/0 or 4/0) of the *TRP1* marker indicated the presence of both the *CLB2*::*TRP1* and the *SML1*::*TRP1* disruption genes in the mutant spore. In addition, all spores were examined for the elongated phenotype of *clb2*Δ.

The number of independent mutants tested is indicated in Table S1 in File S1. All mutants with the same genotype were found to behave similarly unless specified.

### Variability of poor growth mutants

A variability of up to 10-fold of one or two mutants over a set of six or seven poor growth mutants was occasionally observed and is not unexpected given the high rates of mutations and chromosome rearrangements that occur in these mutants (Von Borstel *et al.* 1971). This variability did not qualitatively change the discussed interaction and is addressed in the *Results* section and in File S1.

### Assaying sensitivity during chronic exposure to HU

To measure viability during chronic exposure to HU, a spot assay was performed. Saturated overnight cultures were serially diluted (1:10), and each dilution, starting with undiluted culture, was spotted out onto yeast extract peptone dextrose (YPD) plates with or without HU at the indicated concentrations. Of note, residual growth at the most concentrated cell densities was observed for all mutants, most likely owing to the time taken for HU to penetrate the cells. To allow an easier comparison of sensitivity, in some repeated experiments, if a mutant was observed to grow poorly, the cultures were concentrated (by spinning the culture at 3400 tr/min for 10 min and removing part of the YPD before suspension) to give approximately the same number of cells for all mutants. Alternatively, more cells were inoculated initially. Plates were incubated at 30° for 2–11 d. Images were taken starting on day 2 and every other day until growth ceased (up to 11 d).

### Epistasis analysis

There are three types of genetic interactions, additive, epistatic, and synergistic. Additive interaction indicates that genes function in independent pathways. Deleterious effects of mutant alleles in independently functioning genes are expected to combine multiplicatively compared with WT (or mutant background). For example, a 20-fold effect from one gene mutation and a 10-fold effect from another gene mutation (both either increased or decreased with respect to WT at a given HU concentration) would be expected to result in a 200-fold effect compared with WT. The interaction is called epistatic if the phenotypic impact of the double mutation is less than expected (equal to one of the single mutations in an extreme case). An epistatic interaction indicates that genes function within a common pathway or complex. Finally, a synergistic interaction, when the phenotypic impact of the double deletion is greater than predicted, is interpreted to reflect the existence of parallel (redundant or compensatory) pathways on a common substrate (Avery and Wasserman 1992; Boone *et al.* 2007; St Onge *et al.* 2007). Synergism between two genes does not exclude the possibility that they may also function in the same pathway.

### Data availability

Strains are available upon request. File S1 contains detailed description of all supplemental files, including one table and 12 supplemental figures. Table S1 in File S1 contains the list of strains used in this study and the number of independent mutants tested. Figures S1–S12 in File S1 contain additional mutants and experiments, and all supplemental legends. The variability among poor growth mutants with the same genotype is also addressed in File S1 and the corresponding supplemental figures.

## Results

### Growth of the clb2Δ mutant is weakly inhibited at high HU concentration

**Figure 2:**
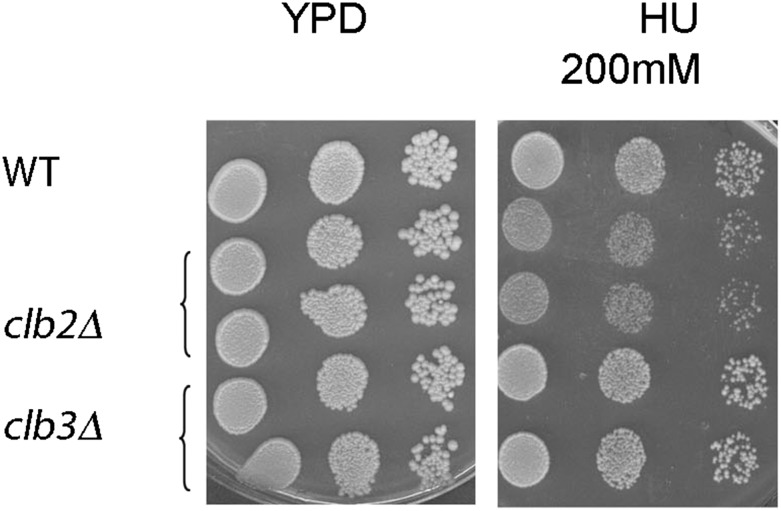
Growth of *clb2*Δ cells is weakly inhibited at elevated HU concentration. Overnight cultures of WT, *clb2*Δ, and *clb3*Δ mutants were serially diluted 10-fold and spotted on YPD plates, with or without HU at the indicated concentration (from left to right, undiluted to 10-fold serially diluted culture). Plates were photographed after 3 d of growth at 30°. Comparable results were obtained in >5 experiments.

**Figure 3:**
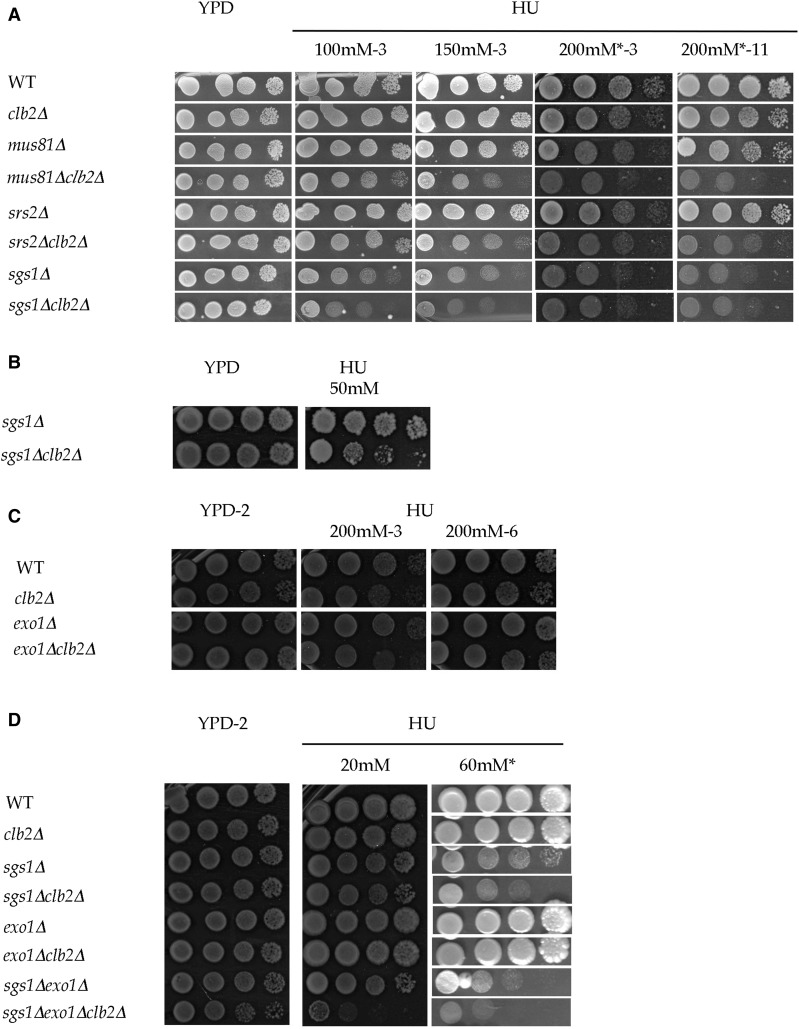
*clb2*Δ has a synergistic effect with *mus81*Δ, *srs2*Δ, and *sgs1*Δ and an additive effect with *exo1*Δ. (A) *clb2*Δ has a synergistic effect with *mus81*Δ and *srs2*Δ. * indicates separate experiment; the YPD plate looked similar to the shown YPD plate. Comparable results were obtained in five independent experiments. (B) *clb2*Δ has synergistic effect with *sgs1*Δ. The HU plate was photographed after 7 d of growth. Comparable results were obtained in >5 experiments. (C) *CLB2* has an additive interaction with *EXO1*. Comparable results were obtained in >5 experiments. (D) Genetic interactions between *CLB2*, *SGS1*, and *EXO1* in WT cells. HU plates were photographed after 5 and 6 d of growth at 30°. * indicates separate experiment; the YPD plate looked similar to the shown YPD plate. Comparable results were obtained in four independent experiments.

In order to investigate whether mitotic cyclins were involved in the replication process, the sensitivity to chronic exposure to HU for the WT, *clb2*Δ, and *clb3*Δ strains, deleted for the principal mitotic cyclin gene *CLB2* or another mitotic cyclin gene *CLB3*, was examined using a spot-dilution assay. The number of independent mutants tested is indicated in Table S1 in File S1. The data presented in Figure 2 show that the growth of *clb2*Δ cells was only mildly inhibited at high HU concentrations (200 mM), whereas *clb3*Δ cells growth was similar to that of the WT strain. At lower HU concentrations, after 2–3 d of growth, the growth of the *clb2*Δ mutant was found to be similar to that of the WT cells (Figure 3A). This mild *clb2*Δ-induced phenotype could be due, however, to the complementary activity of the other mitotic cyclins, and mitotic cyclin–Cdk1 activity could actually have an important role in the late replication phase, since Clb2 expression starts at the end of S phase. Then, in association with the deletion of genes involved in fork stability and restart, full mitotic activity might be required owing to the presence of a high number of stalled forks and the need for fully functional alternative pathways. By measuring the phenotypic impact associated with one mutation in the presence of a second gene mutation, it is possible to define genes functioning within common (epistatic interaction) or parallel (synergistic interaction) pathways (see *Epistasis analysis* in *Materials and Methods*) and to infer regulatory hierarchies or functional complexes (Avery and Wasserman 1992; Boone *et al.* 2007; St Onge *et al.* 2007). Given that *clb2*Δ cells display only a mild growth defect at 200 mM HU, the effect of associating *clb2*Δ with any genes that function in an independent pathway (additive interaction) is expected to be a mild growth defect on top of the effect of the associated gene deletion when plated at HU 200 mM, and no effect at lower HU concentrations.

### CLB2 has synergistic interaction with MUS81, SGS1, and SRS2 and additive interaction with EXO1 during chronic exposure to HU

Mus81 is a structure-specific endonuclease that cleaves replication fork-like structures, nicked Holliday junctions, D-loops, and 3′ flaps (Boddy *et al.* 2001; Kaliraman *et al.* 2001; Doe *et al.* 2002; Fabre *et al.* 2002). Its activity is normally restricted to G2/M or mitosis and is regulated by mitotic cyclin activity (Matos *et al.* 2011; Gallo-Fernández *et al.* 2012). *mus81*Δ mutants accumulate recombination intermediates during replication (Doe *et al.* 2002; Fabre *et al.* 2002). The *mus81*Δ*clb2*Δ and *sgs1*Δ*clb2*Δ mutants were originally constructed (Signon and Simon 2014) because Mus81 had been identified as a Clb2 substrate (Uetz *et al.* 2000). *MUS81* functions in parallel to *SGS1* during challenged and unchallenged replication, and *sgs1*Δ*mus81*Δ is synthetic lethal (Kaliraman *et al.* 2001; Mullen *et al.* 2001; Fabre *et al.* 2002). As shown in Figure 3A, the growth of the *mus81*Δ mutant was not affected at 150 mM HU and mildly inhibited at 200 mM HU, similar to the growth of the *clb2*Δ cells (Figure 3A and Figure S1, A and B in File S1). The expectation for independent functions of these genes would be no effect on growth at 150 mM HU and a stronger growth inhibition at 200 mM HU, with at most a 10-fold drop in plating efficiency for the double *mus81*Δ*clb2*Δ mutants. Surprisingly, though, whereas neither *mus81*Δ nor *clb2*Δ cells exhibited cell death at 200 mM HU, deleting *CLB2* in *mus81*Δ cells resulted in complete cell death and strong growth inhibition at and below this concentration. Indeed, a decrease in plating efficiency of ∼50-fold at 150 mM HU and a decrease of at least 1000-fold at 200 mM HU was observed for the *mus81*Δ*clb2*Δ mutants (Figure 3A and Figure S1, A and B in File S1). Whereas *mus81*Δ cells continued to grow between 3 and 11 d at 200 mM HU (200 mM-3, 200 mM-11, Figure 3A), *mus81*Δ*clb2*Δ cells did not. The growth observed for *mus81*Δ*clb2*Δ cells at the most concentrated cell densities corresponded to a few rounds of cell division (most likely owing to the time taken for HU to penetrate the cells) before cell death (see *Assaying sensitivity during chronic exposure to HU* in *Materials and Methods*). A synergistic effect indicates that *CLB2* and *MUS81* function on a common substrate in parallel pathways, and the strength of the effect reflects an important role of *CLB2* in this pathway (and is also correlated to the role, preponderant or not, of this alternative pathway). Thus, this result suggests that *CLB2* has an important role in the dissolution of fork junctions or recombinant structures, when replication is slow, in the absence of the Mus81 resolvase. Alternatively, or in addition, the absence of Clb2 might lead to increased formation of fork junctions or recombinant structures that require processing by Mus81. This synergistic effect with *mus81*Δ supports the idea that Clb2 regulates the helicase Sgs1 (Signon and Simon 2014) that dissolves fork-like and recombinant structures in parallel to Mus81, in response to HU.

In addition to its role in resolving fork-like structures and Holliday junctions, the helicase Sgs1 prevents nuclease activity and ssDNA accumulation at the stalled fork to avoid fork collapse during replication (Fabre *et al.* 2002). In addition, DNA polymerase is not stably associated with the forks in *sgs1*Δ cells in response to HU treatment (Cobb *et al.* 2003). The growth of the *sgs1*Δ mutant was strongly inhibited in response to HU (>100 mM), and *sgs1*Δ cells displayed cell death at elevated HU concentrations. The growth of the *sgs1*Δ*clb2*Δ mutant appeared only slightly more inhibited than that of the single *sgs1*Δ mutant at HU concentrations ranging from 100 to 200 mM (Figure 3A and Figure S1, A and B in File S1). However, at lower HU concentrations, at which *sgs1*Δ cells could form regular colonies (∼50 mM Figure 3B and Figure S2A in File S1), *clb2*Δ was clearly observed to mildly enhance the sensitivity and cell death of *sgs1*Δ cells by ∼20–50-fold (Figure 3B and Figure S2A in File S1). Thus, in contrast to the epistatic interaction observed in response to MMS (Signon and Simon 2014), deleting *CLB2* has a synergistic effect with *sgs1*Δ in response to HU. Although this result does not exclude the possibility that Clb2 could also regulate Sgs1 in response to HU, this synergistic effect indicates that *CLB2* has a function parallel to that of *SGS1*. Thus, Clb2 could be involved in the dissolution of fork junctions, preventing nuclease activity at stalled forks, or replisome stability. The idea, however, that this synergistic effect is due to the function of Clb2 in the dissolution of fork junctions by regulating Mus81 (Gallo-Fernández *et al.* 2012), although not excluded, is hard to reconcile with the fact that deleting *CLB2* in *sgs1*Δ cells has no effect in response to MMS (Signon and Simon 2014). This result indicates that the absence of *CLB2* alone does not cause significant defects in Mus81 activity. Otherwise, a synergistic effect would have been found between *SGS1* and *CLB2* in response to MMS, since *sgs1*Δ*mus81*Δ is synthetic lethal (Mullen *et al.* 2001). This, then, suggests that the other mitotic cyclin–Cdk1 complexes are able to provide a sufficient level of Mus81 activity in response to MMS. Presumably, this is the case in the response to HU (in which, moreover, *MUS81* plays a minor part, since *mus81*Δ cells do not show cell death at 200 mM HU). This, then, suggests that the synergistic effect of *clb2*Δ with *sgs1*Δ in response to HU is due to a role of Clb2 in preventing nuclease activity at stalled forks or in replisome stability or function in parallel with that of Sgs1.

The helicase Srs2 prevents HR during S phase (Lee *et al.* 1999; Krejci *et al.* 2003; Veaute *et al.* 2003; Pfander *et al.* 2005). The combination of *srs2*Δ with *sgs1*Δ leads to poor cell growth and lethality, owing to hyper-recombination and formation of toxic recombinant structures (Gangloff *et al.* 2000). Although *srs2*Δ and *clb2*Δ cells grew nearly as well as WT cells at 200 mM HU, the growth of the *srs2*Δ*clb2*Δ mutant was strongly inhibited at and below this concentration and displayed sensitivity and cell death at 200 mM HU (Figure 3A and Figure S1, A and B in File S1). Since removing *SRS2* favors the HR pathway, this synergistic effect suggests an important role of Clb2 in the HR pathway. Given that *srs2*Δ cells are not sensitive to HU, favoring the HR pathway, by itself, does not lead to HU sensitivity. This suggests that the absence of Clb2 leads to the formation of toxic recombinant structures when replication is slow. This result also supports the idea that Clb2 could regulate Sgs1 helicase in response to HU.

The exonuclease *EXO1* shows a strong genetic interaction with *SGS1* in the DSB repair pathway and in response to MMS (Mimitou and Symington 2008; Zhu *et al.* 2008; Signon and Simon 2014). In response to HU, the growth of the *exo1*Δ mutant resembles that of the WT at 200 mM HU (Figure 3C and Figure S1B in File S1). This suggests that Exo1 has a minor or no role during HU response in WT cells and is consistent with the inhibition of Exo1 activity by checkpoint function (Cotta-Ramusino *et al.* 2005; Segurado and Diffley 2008; Engels *et al.* 2011). As expected for independent functions of *CLB2* and *EXO1* in response to HU, the growth of *exo1*Δ*clb2*Δ cells was found to be similar to that of the *clb2*Δ mutant (Figure 3C and Figure S1B in File S1), consistent with the lack of effect of *exo1*Δ and the mild growth defect of *clb2*Δ.

Thus, although these data do not exclude the possibility that *CLB2* could also regulate the associated gene, they show that in response to chronic exposure to HU, *CLB2* functions in parallel with *MUS81*, *SRS2*, and *SGS1*. These results suggest that Clb2 is involved at stalled forks in the dissolution of fork junctions and the formation of recombinant structures, and support the idea that Clb2 could regulate Sgs1 in these two processes. In addition, Clb2 functions in parallel with Sgs1 in preventing nuclease activity at stalled forks or in replisome stability in response to HU.

### *clb2*Δ has a dramatic effect with *sgs1*Δ *exo1*Δ

I next tested the sensitivity of a mutant carrying combined deletions of *SGS1*, *EXO1*, and *CLB2* (Signon and Simon 2014). Although the deletion of *EXO1* did not affect cell viability in response to 200 mM HU (Figure 3C), it mildly increased the sensitivity ∼50-fold of the *sgs1*Δ cells to 60 mM HU (Figure 3D and Figure S2A in File S1). This synergistic effect suggests that Sgs1 and Exo1 function in parallel in response to HU, which is consistent with their redundant activity in DNA end resection. Deleting *CLB2* greatly increased the HU sensitivity of double *sgs1*Δ*exo1*Δ cells, as *sgs1*Δ*exo1*Δ*clb*2Δ mutants lost viability even at a very low HU concentration (20 mM). Growth of the triple mutant was impaired even on YPD plates without HU (Figure 3D and Figure S2, A and B in File S1). This strong synergistic effect indicates that *SGS1–EXO1* and *CLB2* function in parallel pathways during replication and are essential for proper fork progression, when the dNTP pool is only mildly decreased. Given that Exo1 and Sgs1 have been shown to act redundantly in DNA end resection (Mimitou and Symington 2008; Zhu *et al.* 2008), this suggests that Clb2 regulates DNA resection and the activities of other nucleases and helicases that are essential for fork stability and progression in the absence of Sgs1 and Exo1.

### CLB2 has synergistic interaction with HR genes RAD52 and RAD51 in response to chronic exposure to HU

HR proteins have a conserved essential role in fork protection and replication restart, besides their role in DSB repair, as reported by works carried out in bacteria, yeast, and mammals. Yeast and mammalian Rad51 protein plays a central part in HR by forming nucleoprotein filaments on ssDNA to perform the homology search and strand exchange reaction (Baumann *et al.* 1996; Mortensen *et al.* 1996; Symington *et al.* 2014). During replication, Rad51 protects the newly replicated strand from extensive resection by nucleases (Hashimoto *et al.* 2010; Petermann *et al.* 2010; Schlacher *et al.* 2011) and participates in the stabilization and reloading of replisome components at stalled forks (Hashimoto *et al.* 2011; Zeman and Cimprich 2014). HR is crucial for the restart of stalled and collapsed forks, as it catalyzes template switching (Figure 1), thus preventing DSB formation (Saintigny *et al.* 2001; Lambert *et al.* 2010; Petermann *et al.* 2010; Carr and Lambert 2013). HR mutants are extremely sensitive to HU and show increased DSB formation in mammals (Saintigny *et al.* 2001; Petermann *et al.* 2010). In budding yeast, HR depends on the *RAD52* epistasis group that includes *RAD51* (Mortensen *et al.* 1996; Symington *et al.* 2014).

**Figure 4:**
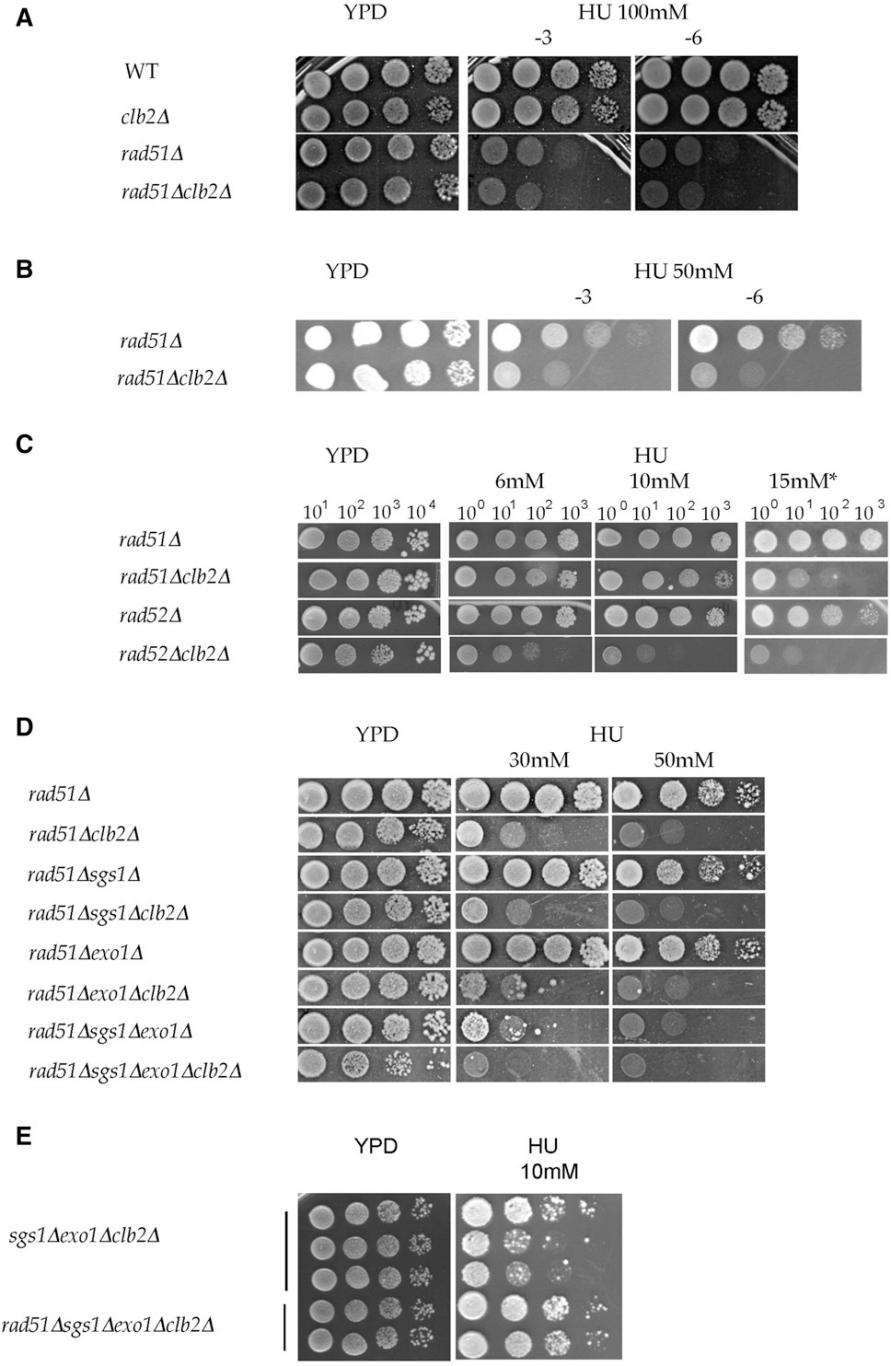
*clb2*Δ has a strong synergistic effect with *rad51*Δ and *rad52*Δ. (A) Sensitivity of WT, *clb2*Δ, *rad51*Δ, and *rad51*Δ*clb2*Δ cells at 100 mM HU concentration. The plate was photographed after 3 d (−3); and after 6 d (−6) of growth at 30°. (B) *clb2*Δ has a synergistic effect with *rad51*Δ. (C) *clb2*Δ has a synergistic effect with *rad51*Δ and *rad52*Δ. Plates were photographed after 3 d of growth. * indicates separate experiment; the YPD plate looked similar to the shown YPD plate. Comparable results were obtained in >5 independent experiments. (D) Genetic interactions between *RAD51*, *CLB2*, *SGS1*, and *EXO1*. HU plates were photographed after 7 d of growth. Comparable results were obtained in three independent experiments. (E) *exo1*Δ*sgs1*Δ*clb2*Δ cells are deficient for (proper) *RAD51* HR. Comparable results were obtained in four independent experiments. * indicates independent experiments, YPD plate looked similar.

As shown in Figure 4A, *rad51*Δ and *rad51*Δ*clb2*Δ cells did not grow at 100 mM HU. Lowering the HU concentration to 50 mM or less (down to ∼15 mM) allowed growth of *rad51*Δ cells but not *rad51*Δ*clb2*Δ cells (Figure 4, B and C and Figure S3, A and B in File S1). Similarly, the *rad52*Δ*clb2*Δ mutants were much more sensitive than the *rad52*Δ mutants. At 10 mM HU, a drop of ∼1000-fold (five mutants out of seven) or 100-fold (two mutants out of seven) in plating efficiency was observed for *rad52*Δ*clb2*Δ mutants compared with *rad52*Δ mutants (see *Variability of poor growth mutants* in *Materials and Methods* and Figure S3, B–D in File S1). At 15–18 mM HU, all the *rad52*Δ*clb2*Δ mutants displayed a drop of at least 1000-fold in plating efficiency compared with *rad52*Δ mutants (Figure 4C and Figure S3, B–D in File S1). This strong synergistic effect indicates that *CLB2* has an important role during replication in parallel with *RAD52* and *RAD51* in response to HU. Given that ∼99% of *rad52*Δ cells and 65% of *rad51*Δ cells lose viability in the presence of a single chromosomal DSB (Weiffenbach and Haber 1981; Sugawara *et al.* 1995; Malkova *et al.* 1996), no DSBs are formed below the HU concentration of ∼15 mM for *rad52*Δ, or ∼50 mM for *rad51*Δ mutants. The fact that the double mutants *rad52*Δ*clb2*Δ and *rad51*Δ*clb2*Δ are extremely sensitive at and below these concentrations (Figure 4C) indicates a positive role of Clb2 independent of DSB repair.

The role of Clb2 in chromosome segregation is unlikely, by itself, to explain this strong synergistic effect. First, as shown above, *clb2*Δ mutants do not show cell death at a very high HU concentration (200 mM), indicating correct chromosomes segregation, in line with the previous observation that chromosomes segregate correctly in the absence of DNA damage or after recovery from DNA damage in the *clb2*Δ mutant (Signon 2011). Then, the sensitivity of HR mutants to HU is due to a defect in fork stability and restart, and their chromosome segregation defects are due to incomplete replication and unresolved DNA structure, and not, *per se*, to a direct involvement of HR proteins in chromosome segregation (Gelot *et al.* 2015). The idea that *RAD52* and *RAD51* function in a compensatory pathway to *CLB2* in chromosome segregation is unlikely. The most likely interpretation of the strong synergistic effect of *clb2*Δ in HR mutants is that Clb2 functions in parallel with the primary function of HR proteins, which is to stabilize and restart the forks, and that more forks stall and collapse in the double mutants as compared with single *rad52*Δ and *rad51*Δ mutants. Thus, Clb2 could be preventing nuclease activity at the newly replicated strand in parallel with Rad52 and Rad51, and/or be involved in an alternate pathway of fork restart, such as fork reversal or regression independent of HR mechanisms and/or in replisome stability or function.

### CLB2 and both SGS1 and EXO1 are required for (proper) Rad51-dependent recombination

In order to verify whether the deleterious effect of *clb2*Δ in the *rad51*Δ mutant was dependent upon Sgs1 and/or Exo1, combined deletions of *SGS1*, *EXO1*, and *CLB2* were introduced into the *rad51*Δ context (Signon and Simon 2014). As shown in Figure 4D (Figures S4 and S5A in File S1) deleting *CLB2* in *rad51*Δ*sgs1*Δ, *rad51*Δ*exo1*Δ, or *rad51*Δ*exo1*Δ*sgs1*Δ increased cell death, indicating that the negative effect of *clb2*Δ in the *rad51*Δ context is not due (or not only due) to *SGS1* and/or *EXO1*. In this study, the sensitivity of a *rad51*Δ*exo1*Δ*sgs1*Δ*clb2*Δ mutant was compared with that of an *exo1*Δ*sgs1*Δ*clb*2Δ mutant in order to verify whether HR occurred in an *exo1*Δ*sgs1*Δ*clb*2Δ mutant. When plated at HU concentrations ranging from 8 to 15 mM, the four *exo1*Δ*sgs1*Δ*clb2*Δ mutants (Figure S2A in File S1) displayed ∼10-fold differences in sensitivity in equal proportion (2/2). The data show that the *rad51*Δ*exo1*Δ*sgs1*Δ*clb2*Δ mutant had comparable sensitivity to those of two *exo1*Δ*sgs1*Δ*clb*2Δ mutants out of the four, differing by less than five-fold in one experiment (Figure 4E) and without appreciable difference in three experiments (Figure S6, A and B in File S1). However, the growth of the *exo1*Δ*sgs1*Δ*clb*2Δ mutants was much slower, in the presence of HU, (Figure S6C in File S1), suggesting a very mild positive effect of deleting *RAD51* in the *exo1*Δ*sgs1*Δ*clb2*Δ mutant. The two other *exo1*Δ*sgs1*Δ*clb2*Δ mutants were found to be more sensitive by ∼10–50-fold compared with the *rad51*Δ*exo1*Δ*sgs1*Δ*clb*2Δ mutant at HU concentrations ranging from 8 to 15 mM (Figure 4E and Figure S6, A–C in File S1), suggesting a positive effect of removing *RAD51* in the *exo1*Δ*sgs1*Δ*clb2*Δ mutant. Thus, the results suggest that a small or very small number of toxic Rad51-dependent recombinant structures are formed in an *sgs1*Δ*exo1*Δ*clb2*Δ mutant and indicate that proper Rad51-dependent recombination does not occur in an *sgs1*Δ*exo1*Δ*clb2*Δ mutant. Since proper formation of the Rad51 nucleofilament requires adequate DNA resection, these results converge on the idea that Clb2 regulates DNA resection at stalled forks in a redundant pathway to Sgs1 and Exo1 so as to allow proper Rad51 recombination. Thus, these results indicate that Clb2 also functions in the HR pathway in the formation of recombinant structure at stalled forks, as already suggested by the *srs2*Δ*clb2*Δ phenotype. These data further support the idea that Clb2 regulates ssDNA processing and nuclease activity at stalled forks during replication.

### Clb2 is involved in alternate pathways of Okazaki fragment processing

**Figure 5:**
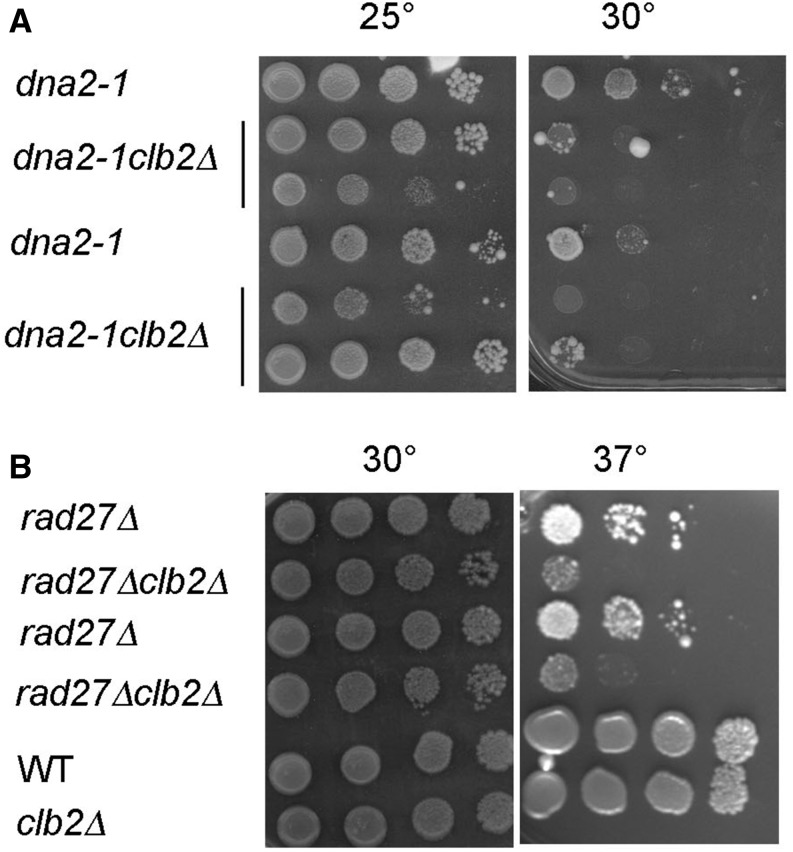
*clb2*Δ increases thermo-sensitivity of (A) *dna2-1* and (B) *rad27*Δ mutants. Comparable results were obtained in four independent experiments.

I next investigated the effect of Clb2 inactivation in two thermo-sensitive nuclease mutants involved in Okazaki fragment processing, *dna-2-1* and *rad27*. Lagging strand DNA replication requires Okazaki fragment processing, including the cleavage of the displaced strand by the flap endonuclease FEN1 (Rad27 in yeast), before it is a few nucleotides long. If the flap extends to a longer length, it allows formation of secondary structure in the ssDNA, which inhibits *FEN1* action, providing a switching mechanism for the processing of the flap between *FEN1* and *DNA2* (Budd *et al.* 1995; Reagan *et al.* 1995; Ayyagari *et al.* 2003). Dna2 is an endonuclease with preference for ssDNA with free ends. Dna2 is phosphorylated by Cdk1 to promote long-range resection at DSB (Chen *et al.* 2011). The viability of *rad27*Δ and *dna2-1* mutants at restrictive temperature depends on HR (Symington 1998; Budd and Campbell 2000). The *dna2-1* mutants displayed variability in their growth and thermo-sensitivity (Figure 5A and data not shown). Two mutants were picked to construct corresponding *dna2-1clb2*Δ mutants. Even though important variability of growth was observed among *dna2-1clb2*Δ mutants, all the *dna2-1clb2*Δ mutants were more thermo-sensitive than the corresponding *dna2-1* mutants at 30° and displayed a decrease in plating efficiency of ∼100-fold (seven out of eight) to 10-fold (one out of eight) (Figure 5A and Figure S7 in File S1). In the *rad27*Δ mutant, deleting *CLB2* increased the thermo-sensitivity ∼50-fold at 37° (Figure 5B). All together, these synergistic effects indicate a role for Clb2 in regulating an alternate pathway of Okazaki fragment processing and further support a role for Clb2 in the processing of ssDNA and the formation of recombinant structures during the replication process.

### clb2Δ increases the viability of mec1Δ, rad53Δ, and rad53K227A checkpoint-deficient cells in response to HU

**Figure 6:**
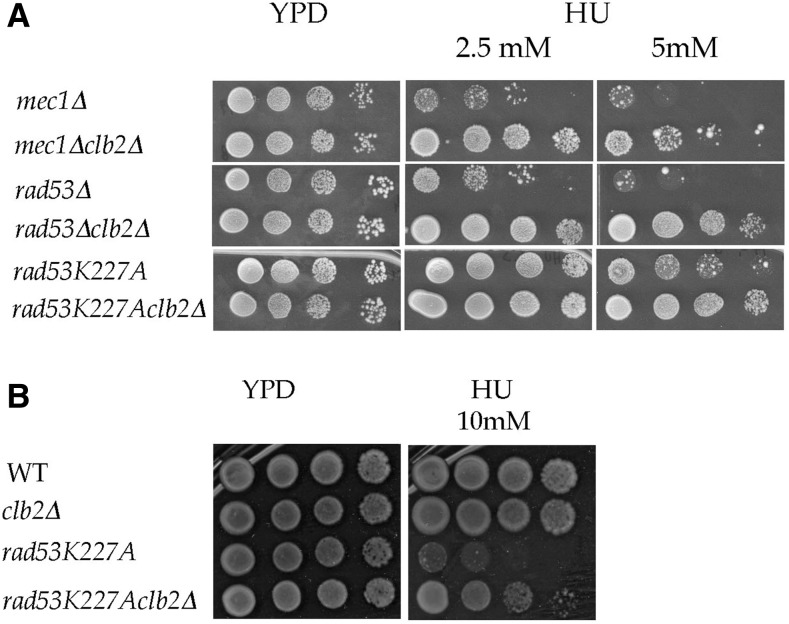
*clb2*Δ improves the viability of *mec1*Δ, *rad53*Δ, and *rad53K227A* to HU (A and B). All checkpoint mutants carry an *SML1* deletion. The plates were incubated at 30° and pictures were taken after 3 d of growth for YPD, 2, 5, and 10 mM HU plates and 4 d for 5 mM HU plates. Comparable results were obtained in >5 experiments.

In the next step, the deletion of *CLB2* was combined with checkpoint-deficient mutants in order to check whether *CLB2* functions in the *MEC1* and/or *RAD53* pathway (Signon and Simon 2014). All checkpoint mutants carry an *SML1* deletion, which rescues the cell lethality of *rad53*Δ and *mec1*Δ cells (Zhao *et al.* 1998). These mutants are extremely sensitive to DNA damaging agents or replication inhibitors (Zhao *et al.* 1998; Friedel *et al.* 2009). Surprisingly, in checkpoint-deficient *mec1*Δ and *rad53*Δ mutants, deleting *CLB2* improved viability ∼100-fold and 1000-fold, respectively, at 5 mM HU (Figure 6A and Figure S8 in File S1), indicating that Clb2 activity is in part responsible for cell death of the *mec1*Δ and *rad53*Δ mutants. A *CLB2* deletion was also introduced into a strain expressing the *rad53K227A* allele, which retains WT *RAD53*-associated growth activity but is deficient for checkpoint function (Fay *et al.* 1997). Deletion of *CLB2* also increased the viability of the *rad53K227A* mutant ∼1000-fold at 10 mM HU (Figure 6, A and B), indicating that in the absence of Rad53 or Rad53 checkpoint function, Clb2 activity is detrimental in response to HU.

Thus, whereas Clb2 activity is mildly beneficial in WT cells (Figure 2), it is deleterious in checkpoint-deficient *mec1*Δ, *rad53*Δ, and *rad53K227A*, suggesting that Clb2 activity is inhibited (or negatively regulated) by Mec1 and Rad53 checkpoint activation in response to HU. This is consistent with the report of Krishnan *et al.*, which showed that Cdc28, in complex with Clb2, remains largely dephosphorylated and shows higher activity in *mec1-1* cells in response to HU.

The positive effect of *clb2∆* is hardly explained by a delay in cell cycle progression that would allow completion of replication, as Krishnan *et al.*, showed that a *mec1-1clb1*Δ*clb2*Δ mutant was only slightly delayed in spindle elongation compared with a *mec1-1* mutant during HU treatment (Krishnan *et al.* 2004). Moreover, given that *mec1*Δ and *rad53*Δ mutants accumulate DSBs in response to HU (Merrill and Holm 1999; Sogo *et al.* 2002; Tercero *et al.* 2003; Friedel *et al.* 2009; Zeman and Cimprich 2014) and that the absence of Clb2 leads to chromosome segregation defects in the presence of a single DSB (Signon 2011), one would expect an increased HU sensitivity of *mec1*Δ and *rad53*Δ cells in the absence of Clb2. An effect of *clb2*Δ on raising the pool of dNTP compared with WT cells is not in agreement with the fact that *clb2*Δ cells display a growth defect at elevated HU concentrations. Since *mec1*Δ and *rad53*Δ cells die of irreversible fork collapse, the most reasonable interpretation for the increased viability of the *mec1*Δ*clb2*Δ, *rad53*Δ*clb2*Δ, and *rad53K227Aclb2*Δ mutants during HU treatment is that the absence of Clb2 decreases the occurrence of fork collapse. These results further support the hypothesis of a role for Clb2 in fork processing.

### The clb2Δ-induced phenotype in mec1Δ, rad53Δ, and rad53K227A mutants depends on both SGS1 and EXO1

**Figure 7:**
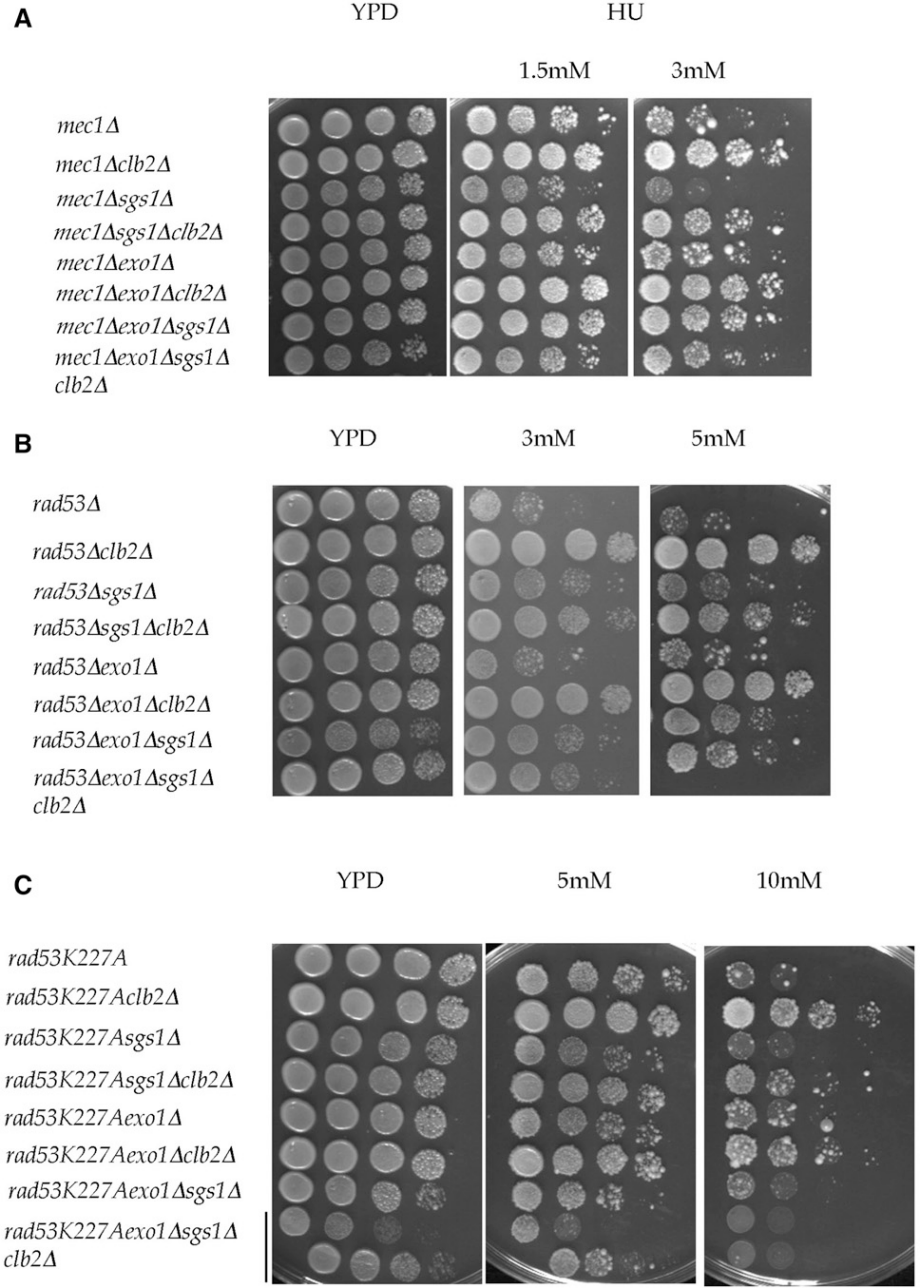
The *clb2*Δ-induced phenotype in checkpoint-deficient cells depends on both *SGS1* and *EXO1*. (A) Checkpoint-deficient *mec1*Δ cells. Plates were photographed after 3 d of growth. Comparable results were obtained in four independent experiments. (B) Checkpoint-deficient *rad53*Δ cells. HU plates were photographed after 3–4 d of growth. Comparable results were obtained in four independent experiments. (C) Checkpoint-deficient *rad53K227A* cells. HU plates were photographed after 3–5 d of growth at 30°. All YPD plates were photographed after 2 d of growth. Comparable results were obtained in four independent experiments.

Combined deletions of *CLB2*, *SGS1*, and *EXO1* were also introduced into checkpoint-deficient cells to verify whether the *clb2*Δ-induced phenotype was dependent upon *SGS1* and/or *EXO1* (Signon and Simon 2014). In *mec1*Δ checkpoint-deficient cells, deleting *SGS1* or *EXO1* had basically no effect on HU sensitivity (Figure 7A and Figure S9, A–C in File S1). The *clb2*Δ-induced rescue phenotype was observed in both *mec1*Δ*sgs1*Δ and *mec1*Δ*exo1*Δ cells. Surprisingly, similar to *CLB2* deletion, deleting both *SGS1* and *EXO1* increased survival to HU of *mec1*Δ cells ∼100-fold at 3 mM HU (Figure 7A and Figure S9, A–C in File S1). This synergistic effect suggests that Sgs1 and Exo1 have a redundant function that impairs the viability of *mec1*Δ cells. Since Sgs1 and Exo1 function redundantly in DNA end resection, presumably some fork collapse is due to deleterious and aberrant Sgs1 and Exo1 resection in the absence of Mec1. Thus, whereas Sgs1–Exo1 is necessary for viability in checkpoint-proficient cells in response to HU, in *mec1*Δ cells its activity is deleterious, suggesting that Mec1 inhibits deleterious Sgs1–Exo1 activity. This is consistent with the role of checkpoint proteins in preventing nuclease activity at stalled forks (Cotta-Ramusino *et al.* 2005; Kai *et al.* 2005; Trenz *et al.* 2006; Froget *et al.* 2008; Segurado and Diffley 2008; Friedel *et al.* 2009; Zeman and Cimprich 2014). The quintuple mutants (+ *sml1*Δ) could not be tested in response to MMS, and have been revealed to be of interest in response to HU. The *mec1*Δ*sgs1*Δ*exo1*Δ*clb2*Δ mutant was found to display sensitivity close to that of the *mec1*Δ*sgs1*Δ*exo1*Δ and *mec1*Δ*clb2*Δ mutants, taking into account the growth on YPD of the *mec1*Δ*sgs1*Δ*exo1*Δ*clb2*Δ mutant compared with *mec1*Δ*exo1*Δ*sgs1*Δ cells (Figure 7A and Figure S9C in File S1). Thus, deleting *CLB2* did not improve further resistance to HU of a *mec1*Δ*sgs1*Δ*exo1*Δ mutant, as would be expected if *CLB2* and *SGS1–EXO1* functioned independently in *mec1*Δ cells. The fact that the effect is less than expected indicates an epistatic interaction and suggests that *CLB2* and *SGS1–EXO1* function in a common pathway in *mec1*Δ cells. The fact that the sensitivity of *mec1*Δ*sgs1*Δ*exo1*Δ*clb2*Δ is close to that of *mec1*Δ*sgs1*Δ*exo1*Δ suggests that the *clb2*Δ-induced rescue phenotype in *mec1*Δ cells depends entirely on both *SGS1* and *EXO1*. This result supports the idea that Clb2 could be regulating a complex formed by Sgs1 and Exo1. Alternatively, Sgs1 and Exo1 could regulate Clb2 activity.

In the *rad53*Δ mutant, deleting *EXO1* did not suppress HU sensitivity (Figure 7B) (Segurado and Diffley 2008). Deleting *SGS1* resulted in a mild improvement of *rad53*Δ viability to HU (Figure 7B and Figure S10 in File S1), although growth on YPD was impeded. Indeed, the *rad53*Δ*sgs1*Δ mutant grew poorly (Figures S10 and S11A in File S1), but its resistance was slightly improved when grown in the presence of HU compared with the *rad53*Δ mutant, suggesting that in the absence of Rad53, the activity of Sgs1 is important for growth but somewhat deleterious under conditions of reduced dNTPs. The *clb2*Δ-mediated sensitivity suppression was observed in both the *rad53*Δ*sgs1*Δ and *rad53*Δ*exo1*Δ contexts, although rescue of *rad53*Δ*sgs1*Δ was slightly less efficient (Figure 7B and Figures S10 and S11, A and B in File S1), suggesting that Sgs1 and Clb2 function in a common pathway and that Sgs1 is in part necessary for the *clb2*Δ-induced phenotype. The combined deletion of *SGS1* and *EXO1* suppressed further sensitivity of *rad53*Δ cells to HU compared with the single *SGS1* deletion, but not to the extent of *clb2*Δ, even though growth was impeded on YPD (Figure 7B and Figure S11, A and B in File S1). This rescue phenotype suggests that, in the absence of Rad53, the activity of Sgs–Exo1 is somewhat deleterious and is responsible for some fork collapse. This suggests that Rad53 inhibits deleterious Sgs1–Exo1 activity in WT cells. This is consistent with the role of checkpoint proteins in inhibiting nuclease activity at stalled forks.

Most interestingly, the *clb2*Δ-mediated sensitivity suppression was abolished in the absence of both *SGS1* and *EXO1*. Indeed, the HU sensitivity of the *rad53*Δ*sgs1*Δ*exo1*Δ*clb2*Δ mutant was similar to that of the *rad53*Δ*exo1*Δ*sgs1*Δ mutant (Figure 7B and Figure S11, A and B in File S1), suggesting that both Sgs1 and Exo1 are necessary for the *clb2*Δ-associated phenotype in *rad53*Δ cells. This supports the idea that Clb2 regulates a complex formed by Sgs1 and Exo1, or combined Sgs1 and Exo1 activity. These results are hard to reconcile with the idea that Sgs1–Exo1 regulates Clb2, given that *clb2*Δ increases viability much more than *exo1*Δ*sgs1*Δ, but the *sgs1*Δ*exo1*Δ*clb2*Δ-induced phenotype resembles the *sgs1*Δ*exo1*Δ-induced and not the *clb2*Δ-induced phenotype in this context. However, it cannot be excluded that Clb2 could function downstream of and require Sgs1–Exo1 activity in the *rad53*Δ mutant.

Contrary to what was observed in the *rad53*Δ mutant, neither *sgs1*Δ nor *sgs1*Δ*exo1*Δ suppressed sensitivity of the *rad53K227A* mutant, and the triple *rad53K227Asgs1*Δ*exo1*Δ cell displayed sensitivity (five mutants out of six; one mutant was more sensitive) comparable to that of *rad53K227Asgs1*Δ, *rad53K227Aexo1*Δ, or *rad53K227A* cells (Figure 7C and Figure S12 in File S1). Thus, although Sgs1 and Sgs1–Exo1 have a deleterious activity in the absence of Rad53, in the presence of Rad53 protein that lacks checkpoint activation but retains growth-associated activity, neither Sgs1 nor Sgs1–Exo1 has a deleterious activity in response to HU. This suggests that Rad53, independently of checkpoint activation, plays a part in preventing deleterious Sgs1 and Sgs1–Exo1 activity at stalled forks. Deleting *CLB2* rescued both the *rad53K227Asgs1*Δ and *rad53K227Aexo1*Δ mutants, although less efficiently than the *rad53K227A* cells (Figure 7C and Figure S12 in File S1), suggesting that *SGS1* and *EXO1* are in part necessary for the *clb2*Δ-induced phenotype.

Most interestingly, *clb2*Δ did not rescue the *rad53K227Asgs1*Δ*exo1*Δ mutant. Instead, the *rad53K227Asgs1*Δ*exo1*Δ*clb2*Δ mutant exhibited HU sensitivity close to that of the *rad53K227Asgs1*Δ*exo1*Δ and *rad53K227A* mutants, although growth on YPD was affected (Figure 7C). This indicates that Sgs1–Exo1 activity is responsible for the *clb2*Δ-associated phenotype in the *rad53K227A* mutant in response to HU, and that Clb2 regulates combined Sgs1 and Exo1 activity or a complex formed by Sgs1 and Exo1. The idea that Clb2 functions downstream of Sgs1–Exo1 or is regulated by Sgs1–Exo1 is difficult to reconcile with the fact that *sgs1*Δ*exo1*Δ has no effect on HU sensitivity in *rad53K227A*, whereas *clb2*Δ has a strong beneficial effect, and that the *sgs1*Δ*exo1*Δ*clb2*Δ-induced phenotype resembles *sgs1*Δ*exo1*Δ-induced and not the *clb2*Δ-induced phenotype.

Thus, in all checkpoint mutants, the *clb2*Δ-induced rescue phenotype depends on both *SGS1* and *EXO1*, although *sgs1*Δ*exo1*Δ behaves differently, strengthening the idea that Clb2–Cdk1 regulates a complex formed by Sgs1 and Exo1, or the combined activity of Sgs1 and Exo1, at stalled forks (Signon and Simon 2014).

**Figure 8:**
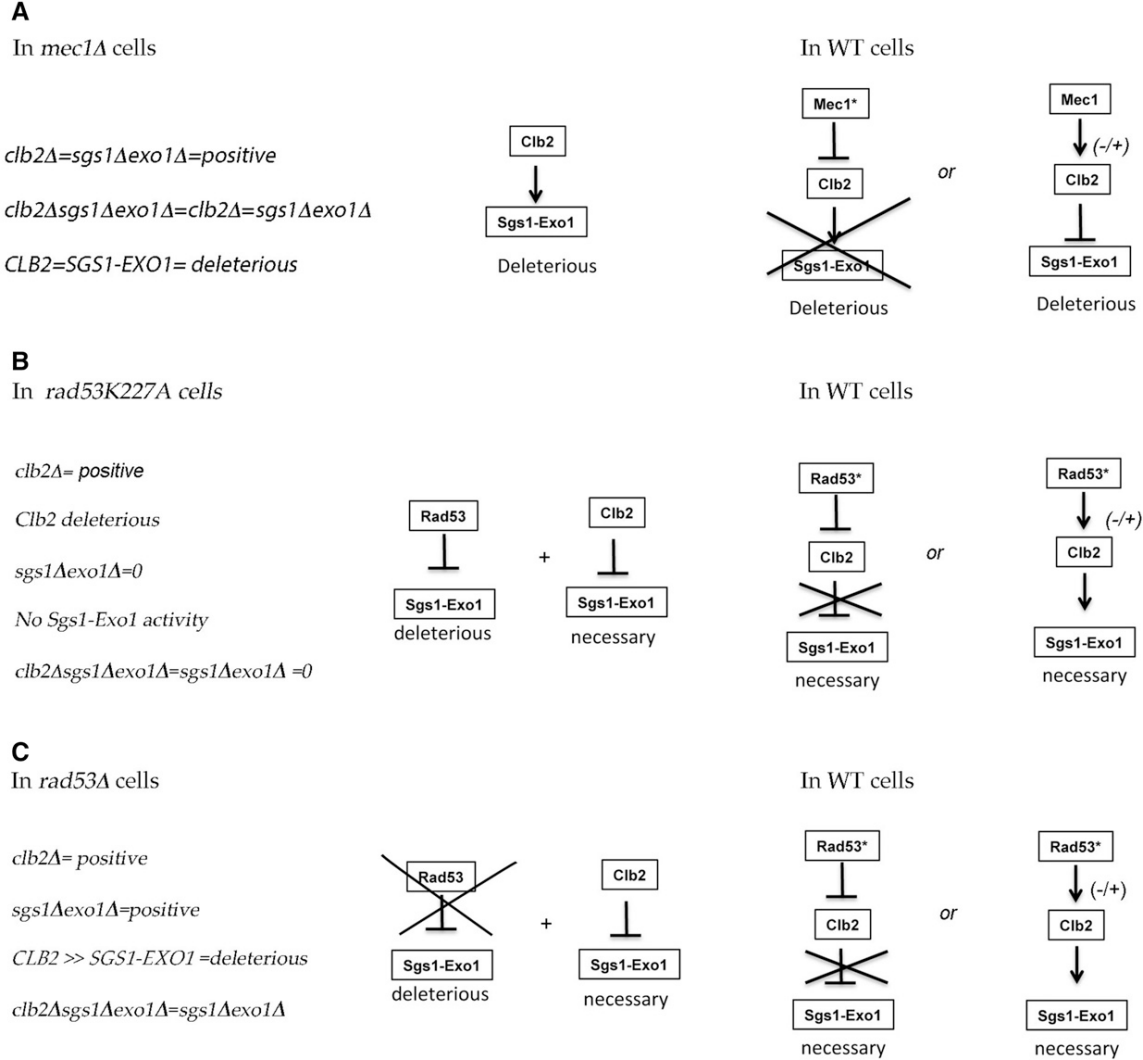
Model for the regulation of Sgs1–Exo1 by Clb2 in a Mec1- and a Rad53-dependent pathway. (A) In *mec1*Δ cells, *clb2*Δ has a positive effect, similar to *sgs1*Δ*exo1*Δ, and *sgs1*Δ*exo1*Δ*clb2*Δ has the same effect as *sgs1*Δ *exo1*Δ. This suggests that Clb2 activity is responsible for the deleterious activity of Sgs1–Exo1. Thus, it suggests that in WT cells, Mec1 inhibits Clb2 activity, thereby preventing deleterious Sgs1–Exo1 activity. Alternatively, the regulation by Mec1 of Clb2 could lead to the inhibition by Clb2 of Sgs1–Exo1 activity, which implies that the regulation by Mec1 switches the Clb2 activity from activation to inhibition of Sgs1–Exo1 activity (see below for discussion of this model). (B) In *rad53K227A*, *sgs1*Δ*exo1*Δ has no effect, suggesting that Rad53, independently from checkpoint activation, inhibits deleterious Sgs1–Exo1 activity. *clb2*Δ has a positive effect and *sgs1*Δ*exo1*Δ*clb2*Δ has no effect, as does *sgs1*Δ*exo1*Δ. Thus, the *clb2*Δ-associated phenotype depends entirely on Sgs1–Exo1, which suggests that *clb2*Δ allows necessary Sgs1–Exo1 activity in *rad53K227A* cells. Thus, it suggests that Clb2 inhibits necessary Sgs1–Exo1 activity in *rad53K227A* cells. This, then, suggests that in WT cells, Rad53 checkpoint activation inhibits Clb2, thereby preventing the inhibition of Sgs1–Exo1 activity. Alternatively, Rad53 checkpoint activation regulates Clb2 to induce Sgs1–Exo1 activity, which implies that the regulation by Rad53 checkpoint activation switches the Clb2 activity from inhibition to activation of Sgs1–Exo1 activity (see below for discussion of this model). (C) In *rad53*Δ cells, the *clb2*Δ-induced phenotype is stronger than the *sgs1*Δ*exo1*Δ-induced phenotype. Sgs1–Exo1 has a deleterious activity, owing to the absence of Rad53, but has a less important phenotype than *clb2*Δ, which inhibits necessary Sgs1–Exo1 activity as suggested above. This fits with the interpretation that in WT cells, Rad53 checkpoint activation inhibits (or regulates) Clb2 to allow necessary Sgs1–Exo1 activity. The model of switch is not suggested by the fact that the absence of Clb2 prevents Sgs1–Exo1 activity in *mec1*Δ cells, and thus that Clb2 is not necessary to inhibit Sgs1–Exo1 activity, but rather its absence leads to a lack of its activity. Similarly, the absence of Clb2 allows Sgs1–Exo1 activity in *rad53K227A* and *rad53* cells, and thus Clb2 is not necessary to activate Sgs1–Exo1 activity, but its absence leads to activation of Sgs1–Exo1. The fact, however, that Clb2 is found to induce Sgs1–Exo1 activity in *mec1*Δ cells and to inhibit Sgs1–Exo1 activity in *rad53* checkpoint-deficient cells agrees with the idea that a switch in Clb2 activity occurs at least in a Mec1-dependent pathway. Indeed, in *rad53*Δ and rad53K227A checkpoint-deficient cells, Mec1 is present and Clb2 is found to inhibit Sgs1–Exo1 activity.

In sum, the deletion of *CLB2* increases viability in *mec1*Δ cells, as does the deletion of *SGS1–EXO1*. Thus, the deleterious activity of Clb2 corresponds to a deleterious activity of Sgs1–Exo1, which suggests that, in *mec1*Δ cells, Clb2 activity is responsible for the deleterious Sgs1–Exo1 activity (Figure 8A). This suggests that, in WT cells, Mec1 inhibits (or regulates) Clb2 activity to prevent deleterious Sgs1–Exo1 activity (Figure 8A).

In both the *rad53K227A* and *rad53*Δ cells, deleting *CLB2* increases viability and does not correspond to deleting *EXO1* and *SGS1* (Figure 8, B and C). *sgs1*Δ*exo1*Δ has no effect in *rad53K227A* and a much smaller effect than the *clb2*Δ-induced rescue phenotype in the *rad53*Δ mutant. Yet, the *clb2*Δ-induced rescue phenotype depends on both *SGS1* and *EXO1*. This suggests that beneficial (necessary) activity of Sgs1–Exo1 occurs in the absence of Clb2 in the *rad53K227A* and *rad53*Δ mutants, and thus that Clb2 inhibits necessary Sgs1–Exo1 activity in the absence of Rad53 checkpoint activation. These results were interpreted to mean that, in WT cells, Rad53 checkpoint activation inhibits (or regulates) Clb2 to allow necessary Sgs1–Exo1 activity (Figure 8, B and C).

Overall, the results presented above suggest that the regulation by Mec1 of Clb2 prevents deleterious Sgs1–Exo1 activity, whereas the regulation by Rad53 checkpoint activation of Clb2 allows necessary Sgs1–Exo1 activity.

## Discussion

### Clb2 is involved in fork stability and restart dependent on and independent of the HR pathway

Given the function of mitotic cyclins, which is to associate with Cdk1 (Cdc28) and provide substrate specificity, the effect of deleting *CLB2* is presumably associated with a defect in Clb2–Cdk1 activity. Aberrant mitotic activity is often found in tumor cells; unraveling the pathways and functions that mitotic activity regulates is crucial. Although roles for mitotic-Cdk1 activity in the HR pathway during DSB repair and the G2/M checkpoint pathway have been well documented recently, this study indicates an unexpected and novel role for Clb2 and mitotic cyclins in the replication process and in response to S phase checkpoint activation. Contrary to mammals, which display fork inactivation and DSB formation after prolonged HU treatment, WT yeast cells treated with 200 mM HU show sustained slow replication progression, and electron micrographs of chromosomes reveal bubble structures that contain obvious stretches of ssDNA but otherwise appear normal (Sogo *et al.* 2002; Feng *et al.* 2006). The temporal program of S phase remains intact but is executed at a much lower pace. Thus, stalled replication forks, induced by HU treatment, are efficiently stabilized and restarted in WT yeast cells, when the pool of dNTPs is low. The mild growth inhibition of *clb2*Δ cells compared with WT cells at 200 mM HU suggests that the absence of Clb2 starts to affect replication, but that the other mitotic cyclins can still overcome the absence of Clb2.

**Figure 9:**
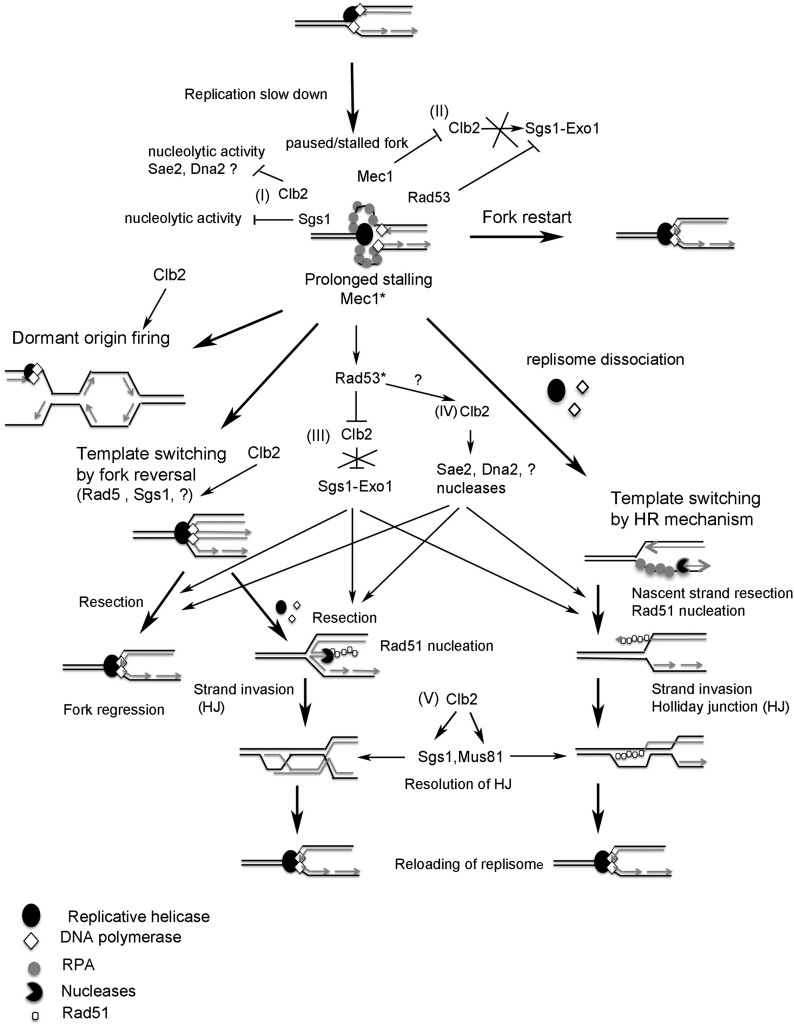
Model for the function and regulation of Clb2 at paused or stalled forks. At paused or stalled forks, Clb2 (I) prevents activity of nucleases such as Sae2 and Dna2. In addition, Mec1 inhibits Clb2 (II), thereby preventing Sgs1–Exo1 activity. Rad53, independently of checkpoint activation, also inhibits Sgs1–Exo1 activity. Replication fork restarts. Prolonged stalling leads to an alternate pathway of fork restart and Mec1 activation. Clb2 is involved in the alternate pathway of fork restart, such as possible new origin firing and/or template switching, dependent on and independent of the HR pathway. Clb2 could regulate Sgs1 and/or Rad5 or unknown (?) proteins involved in promoting fork reversal. Mec1 activation leads to Rad53 checkpoint activation, which regulates Clb2 (III) to allow Sgs1–Exo1 activity. Clb2 regulates the activity of nucleases (IV) such as Sae2 and Dna2 in parallel with Sgs1–Exo1, to promote HR. This process could possibly be regulated by Rad53 checkpoint activation. Finally, Clb2 (V) regulates the resolution of fork junctions and recombinant structures by regulating Sgs1 and Mus81. Possibly, Clb2 is involved in reloading of the replisome.

Of interest, another study also supports a role for *CLB2* in DNA replication (Sclafani *et al.* 2002). Associating *clb2*Δ with genes involved in fork stability or restart and recombination has shed light on functions and pathways regulated by Clb2 and mitotic cyclins during replication. The analysis of each mutant reveals specific function(s) of Clb2 in the considered context, given that defects in fork processing and stalled fork structure differ in each context. The strong synergistic effect of *clb2*Δ with most genes tested reveals an important role for Clb2 in numerous pathways involved in the stability and restart of stalled forks during replication, by both HR-dependent and HR-independent mechanisms. This work indicates that Clb2 functions in the HR pathway at stalled forks during replication and at multiple steps (Figure 9). First, this work indicates that Clb2 is involved in the formation of Rad51-dependent recombinant structures and functions in parallel with Sgs1–Exo1 in this process, suggesting that Clb2 is involved in DNA resection and could positively regulate the activities of Sae2 and Dna2 nucleases at stalled forks that have been recently identified as Clb2 and mitotic Cdk1 substrates during DSB repair (Huertas *et al.* 2008; Chen *et al.* 2011). A role for Clb2 in the formation of recombinant structures during replication is supported by the synergistic effect of *clb2*Δ in the *srs2*Δ mutant. The increased thermo-sensitivity of the *dna2−1clb2*Δ and *rad27*Δ*clb2*Δ mutants at restrictive temperatures further strengthens a role for Clb2 during replication in processing ssDNA and in the formation of recombinant structure. Finally, at a later step, Clb2 is involved in the dissolution of these forks and recombinant structures, as suggested by the *mus81*Δ*clb2*Δ sensitivity. The *clb2*Δ-induced phenotype in the *mus81*Δ, *srs2*Δ, and *dna2-1* mutants supports the idea that Clb2 regulates Sgs1 activity in numerous processes at forks that have been stalled, either by DNA damage as suggested by the previous study (Signon and Simon 2014) or during replication as suggested by this study.

Of great interest, a recent study in mammals demonstrated that mitotic Cdk1 does indeed regulate WRN (the Sgs1 human homolog) at collapsed forks (Palermo *et al.* 2016), indicating that the regulation of RecQ helicase activity by mitotic cyclins has been conserved in higher eukaryotes. This work shows that, during replication, Clb2 also functions in a parallel and redundant pathway to Sgs1 and suggests that Clb2 could prevent nuclease activity or be involved in replisome function at stalled forks. This idea is supported by the synergistic effect of *clb2*Δ in HR mutants, which are also deficient in preventing nucleolytic activity at stalled forks. In this way, Clb2 could prevent Sae2 and Dna2 nuclease activity.

In sum, the data suggest that in some contexts, Clb2 allows nuclease activity to facilitate HR, whereas in others, Clb2 prevents nuclease activity at stalled forks. Although other roles for Clb2 and mitotic Cdk1 during replication are not excluded, this work converges to the idea that *CLB2* controls the activity of numerous nucleases at single-stranded gaps created by DNA replication, including the activity of Sgs1–Exo1 (discussed below). The expression of Clb2 starts at the end of S phase. Interestingly, late DNA replication regions have been defined as RSZs (Cha and Kleckner 2002). Mitotic cyclins could play an important part in the proper replication of these regions that are difficult to replicate.

### Clb2 regulates the combined activity or a complex formed by Sgs1 and Exo1 in a MEC1- and a RAD53-dependent pathway

Nucleases often function in association with helicases that unwind dsDNA (Yeeles and Dillingham 2010). This work further strengthens the idea, suggested by the epistasis analysis in response to MMS (Signon and Simon 2014), that Clb2 regulates the activity of a complex formed by Sgs1 helicase and exonuclease Exo1 at stalled forks. Whereas interactions between the RecQ homolog and Exo1 have been described in human cells in DNA resection during DSB repair (Nimonkar *et al.* 2008, 2011; Aggarwal *et al.* 2010), a recent study, performed in fission yeast, showed that Rqh1 (the fission yeast homolog of Sgs1) constrains Exo1-dependent resection at stalled forks (Osman *et al.* 2016), supporting the idea that Sgs1 and Exo1 also form a complex in yeast, and that Sgs1 is an important regulator of Exo1 activity at stalled forks as suggested by the epistasis analysis in response to MMS (Signon and Simon 2014).

This study reveals novel aspects of Sgs1–Exo1 regulation by Clb2 in Mec1- and Rad53-dependent pathways. These data suggest that whereas the regulation by Mec1 of Clb2 prevents Sgs1–Exo1 activity, the regulation by Rad53 checkpoint activation of Clb2 allows Sgs1–Exo1 activity. The fact that in a *mec1*Δ cell, which displays poor Rad53 checkpoint activation, only the deleterious Sgs1–Exo1 activity is responsible for loss of cell viability is consistent with *MEC1* and *RAD53* having separate functions at the forks (Tercero *et al.* 2003). However, this result also leads to the idea that forks in *mec1*Δ cells collapse before the necessary activity of Sgs1–Exo1 is required and suggests a temporal regulation of Sgs1–Exo1 activity by Mec1 and Rad53. The data fit with the interpretation that Mec1 or Mec1 checkpoint activation first inhibits (or regulates) Clb2–Cdk1 activity to prevent a deleterious Sgs1 and Exo1 activity, then Rad53 checkpoint activation inhibits (or regulates) Clb2–Cdk1 to allow Sgs1 and Exo1 activity. Since it could not be determined whether the phenotype of *clb2*Δ in *mec1*Δ cells was due to the absence of Mec1 or to a defect in checkpoint activation, it is possible that Mec1 could target Clb2–Cdk1 to prevent the nuclease activity of Sgs1–Exo1 at paused forks, prior to and independent of checkpoint activation. Interestingly, the Rad53-associated growth activity is also found to inhibit deleterious Sgs1–Exo1 activity, as suggested by the mild *sgs1*Δ*exo1*Δ-induced rescue phenotype in *rad53*Δ cells and the lack of *sgs1*Δ*exo1*Δ-induced rescue phenotype in *rad53K227A* (Figure 7, B and C and Figure 8, B and C). One model is that Mec1, Clb2, and the Rad53-associated growth activity function independently or in parallel pathways to inhibit deleterious Sgs1–Exo1 activity. A variant of the model is that Mec1, Clb2–Cdk1, and the growth activity of Rad53 might function in the same pathway for this process. Interestingly, the mild negative effect of *clb2*Δ on growth on YPD in *mec1*Δ*exo1*Δ*sgs1*Δ and *rad53K227Aexo1*Δ*sgs1*Δ mutant (both of which have Rad53 protein) contrasts with the mild positive effect in *rad53*Δ*exo1*Δ*sgs1*Δ mutant (Figure 6, A–C) and supports the idea that Rad53 also regulates Clb2 activity during growth, as suggested previously by the MMS study. However, we cannot exclude the idea that it is Mec1 checkpoint activation that regulates Clb2 activity to prevent deleterious Sgs1–Exo1 activity and, later on, Rad53 checkpoint activation that regulates Clb2 to allow necessary Sgs1–Exo1. This is because, interestingly, Mec1 is fully activated by 30 min, whereas Rad53 becomes fully activated by 90 min after HU treatment (Puddu *et al.* 2011), consistent with a temporal regulation of Clb2 by Mec1 checkpoint activation and, subsequently, full Rad53 checkpoint activation.

### Model

One model for the roles of Clb2 and mitotic Cdk1 that accounts for these results could be that, at the end of the S phase, ssDNA is present at stalled forks in specific regions of the chromosome that are difficult to replicate, such as the RSZ (Cha and Kleckner 2002). With ssDNA being the substrate of HR, nuclease activity would first have to be prevented at stalled forks to avoid the possibility of HR that could potentially lead to genomic instability. Clb2–Cdk1 might prevent nuclease/helicase activity at stalled forks by the end of replication (in parallel with Sgs1 and HR), including Sgs1–Exo1 activity dependent on the *MEC1* pathway (dependent on or independent of Mec1 checkpoint activation and the growth activity of Rad53) [Figure 9 (I), (II)], consistent with the effect of *clb2*Δ with *rad52*Δ, *rad51*Δ, *sgs1*Δ, and *mec1*Δ. Then, if replication is not able to restart and is stalled for a prolonged period, activated Mec1, leading to Rad53 activation, would inhibit (or regulate) Clb2 to permit Sgs1–Exo1 activity [Figure 9 (III)]. In addition, Clb2, dependent on or independent of checkpoint activation, allows the activity of other nucleases such as Sae2 or Dna2 [Figure 9 (IV)] to allow resection and alternate pathways of fork restart, such as the HR pathway or fork regression independent of the HR pathway, consistent with the effect of *clb2*Δ with *rad52*Δ, *rad51*Δ, *srs2*Δ, *sgs1*Δ*exo1*Δ, *rad53*Δ, and *rad53K227A*. Finally, Clb2–Cdk1 activity would regulate the dissolution of these recombinant structures by regulating Sgs1, consistent with the synergistic effect of *clb2*Δ with *mus81*Δ, and most likely by regulating the already identified Clb2 substrate Mus81 as well [Figure 9 (V)]. Mitotic cyclin activity would then initiate mitosis, thereby tightly linking replication status to chromosome segregation.

In summary, this work indicates important and numerous roles for Clb2 and mitotic cyclins in the replication process and suggests that mitotic cyclins play an essential part in the stability and restart of paused or stalled forks by modulating the activity of numerous nucleases at single-stranded gaps created by DNA replication. This work provides new perspectives on the role of mitotic cyclins in late replication by the end of S phase.

## Supplementary Material

Supplemental material is available online at www.g3journal.org/lookup/suppl/doi:10.1534/g3.117.300537/-/DC1.
