# Supplementary material for: Genetic Evidence for Roles of Yeast Mitotic Cyclins at Single-Stranded Gaps Created by DNA Replication
Source: G3 (Bethesda). 2017 Dec 26;8(2):737–52. doi: 10.1534/g3.117.300537 (PMC5919743; doi:10.1534/g3.117.300537)
Supplement: Supplementary file 1 [file 737FileS1.pdf]

Table S1

Strain name	relevant genotype	Nber mut	reference
BF264-J15DU	*Mata leu2 ura3 trp1 his2 ade1*		Signon and Simon 2014
YLS37	*Mata leu2 ura3 trp1 his2 ade1,bar-*		Signon and Simon 2014
YLS38	*clb2::URA3, FOAr*	5	Signon and Simon 2014
YLS274	*exo1::URA3*	5	Signon and Simon 2014
YLS276	*exo1::URA3, clb2::LEU2*	6	Signon and Simon 2014
YLS173	*mec1::KAN, sml1::TRP1*	5	Signon and Simon 2014
YLS185	*mec1::KAN, sml1::TRP1, clb2::LEU2*	6	Signon and Simon 2014
YLS236	*mec1::KAN, sml1::TRP1, sgs1::URA3*	5	Signon and Simon 2014
YLS239	*mec1::KAN, sml1::TRP1, clb2::LEU2, sgs1::URA3,*	5	Signon and Simon 2014
YLS266	*mec1::KAN, sml1::TRP1, exo1::URA3*	6	Signon and Simon 2014
YLS269	*mec1::KAN, sml1::TRP1, clb2::LEU2, exo1::URA3*	5	Signon and Simon 2014
YLS732	*mec1::KAN, sml1::TRP1, sgs1::LEU2, exo1::URA3*	4	Signon and Simon 2014
	mec1::KAN, sml1::TRP1, sgs1::LEU2, exo1::URA3, clb2::TRP	4	This study
YLS106	*mus81::TRP1*	2	Signon and Simon 2014
YLS108	*mus81::TRP1, clb2::URA3*	2	Signon and Simon 2014
YLS64	*rad51::KAN*	5	Signon and Simon 2014
YLS72	*rad51::KAN, clb2::TRP1*	5	Signon and Simon 2014
YLS337	*rad51::KAN, sgs1::LEU2*	4	Signon and Simon 2014
YLS340	*rad51::KAN, sgs1::LEU2, clb2::URA3*	4	Signon and Simon 2014
	rad51::KAN, sgs1::LEU2, exo1::URA3	4	Signon and Simon 2014
	rad51::KAN, sgs1::LEU2, exo1::URA3, clb2::TRP	2	Signon and Simon 2014
YLS89	*rad52::TRP*	7	This study
YLS92	*rad52::TRP, clb2::URA3*	7	This study
YLS177	*rad53::NAT, sml1::TRP1*	5	Signon and Simon 2014
YLS188	*rad53::NAT, sml1::TRP1, clb2::TRP*	6	Signon and Simon 2014
YLS230	*rad53::NAT, sml1::TRP1, sgs1::URA3*	5	Signon and Simon 2014
YLS233	*rad53::NAT, sml1::TRP1, clb2::LEU2, sgs1::URA3*	5	Signon and Simon 2014
YLS290	*rad53::NAT, sml1::TRP1,exo1::URA3*	5	Signon and Simon 2014
YLS293	*rad53::NAT, sml1::TRP1, clb2::LEU2,exo1::URA3*	5	Signon and Simon 2014
YLS738	*rad53::NAT, sml1::TRP1,sgs1::LEU2,exo1::URA3*	2	Signon and Simon 2014
	rad53::NAT, sml1::TRP1,sgs1::LEU2,exo1::URA3, clb2::TRP	2	This study
YLS199	*rad53K227A, sml1::TRP1*	5	Signon and Simon 2014
YLS214	*rad53K227A, sml1::TRP1, clb2::LEU2*	5	Signon and Simon 2014
YLS739	*rad53K227A, sml1::TRP1, exo1::URA3*	6	Signon and Simon 2014
YLS741	*rad53K227A, sml1::TRP1,exo1::URA3,clb2::LEU2*	6	Signon and Simon 2014
YLS744	*rad53K227A, sml1::TRP1, sgs1::URA3*	5	Signon and Simon 2014
YLS747	*rad53K227A, sml1::TRP1, sgs1::URA3, clb2::LEU2*	4	Signon and Simon 2014
YLS750	*rad53K227A, sml1::TRP1, exo1::URA3,sgs1::LEU2*	6	Signon and Simon 2014
	rad53K227A, sml1::TRP1, exo1::URA3,sgs1::LEU2,clb2::TRP	3	This study
YLS70	*sgs1::LEU2*	6	Signon and Simon 2014
YLS80	*sgs1::LEU2, clb2::URA3*	6	Signon and Simon 2014
YLS754	*sgs1::LEU2, exo1::URA3*	4	Signon and Simon 2014
	sgs1::LEU2, exo1::URA3, clb2::TRP	4	Signon and Simon 2014
YLS210	*srs2::LEU2*	3	Signon and Simon 2014
YLS212	*srs2::LEU2, clb2::TRP1*	3	Signon and Simon 2014
YLS263	*Rad27::TRP1*	4	Signon and Simon 2014
YLS272	*Rad27::TRP1,clb2::URA3*	4	Signon and Simon 2014
YLS329	*dna2-1*	4	This study
YLS332	*dna2-1,clb2::URA3*	8	This study

List of strains used in this study. First column represents genotype. All mutant strains are isogenic to YLS37. Second column represents number of transformants and/or spores tested.

A

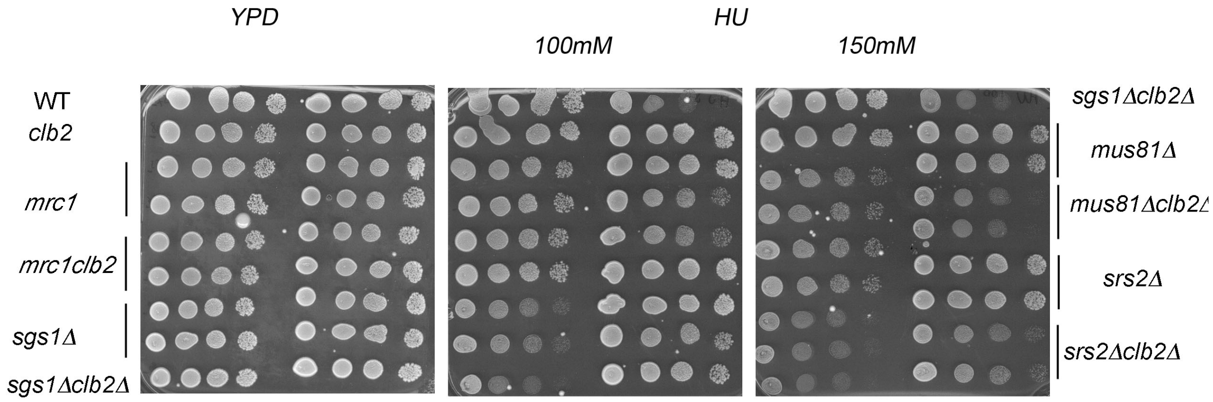

B

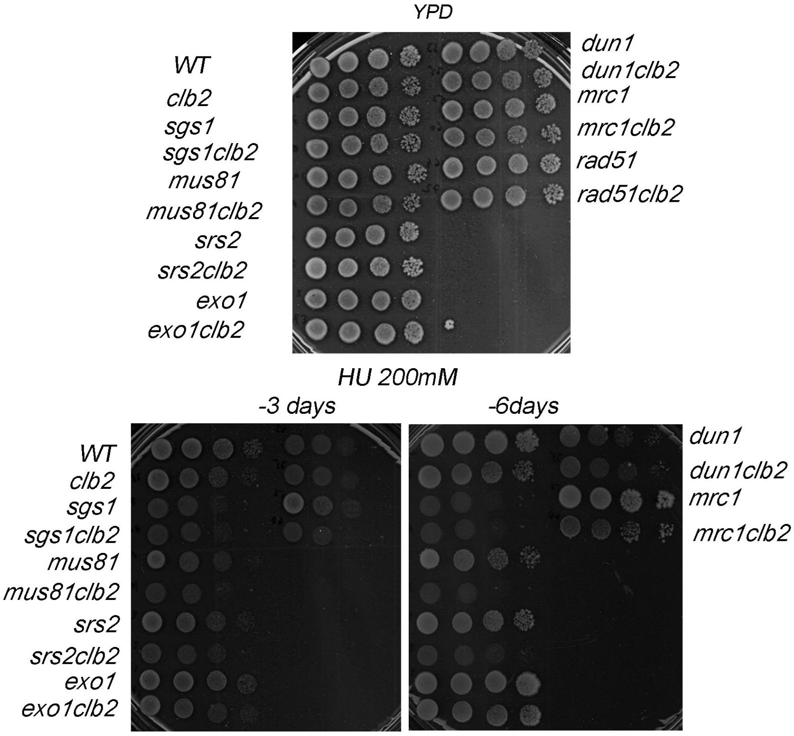

Figure S1. Additional mutants and unstiches images of (A) Figure 3A and (B) Figure 3A and Figure 3C. rad51 and rad51 mutants were not plated on HU 200mM.

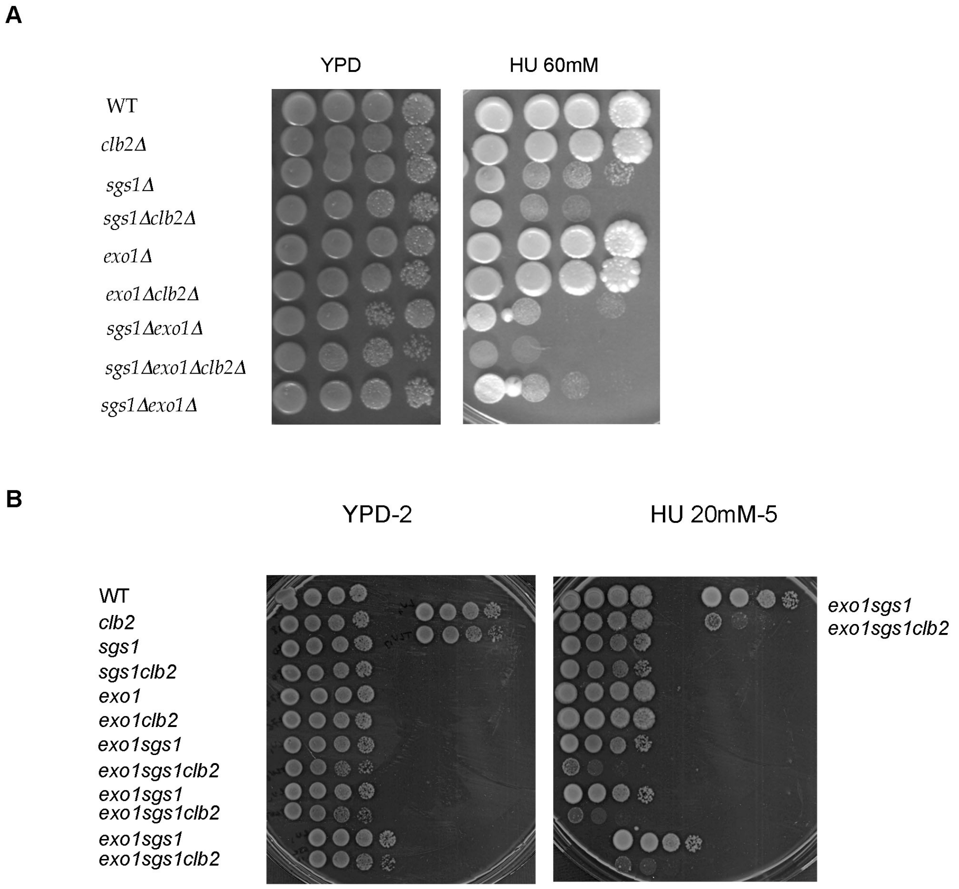

Figure S2. (A) unstiches image of Figure 3D, 60mM HU and corresponding YPD plate. (B) Additional *exo1sgs1* and *exo1sgs1clb2* mutants.

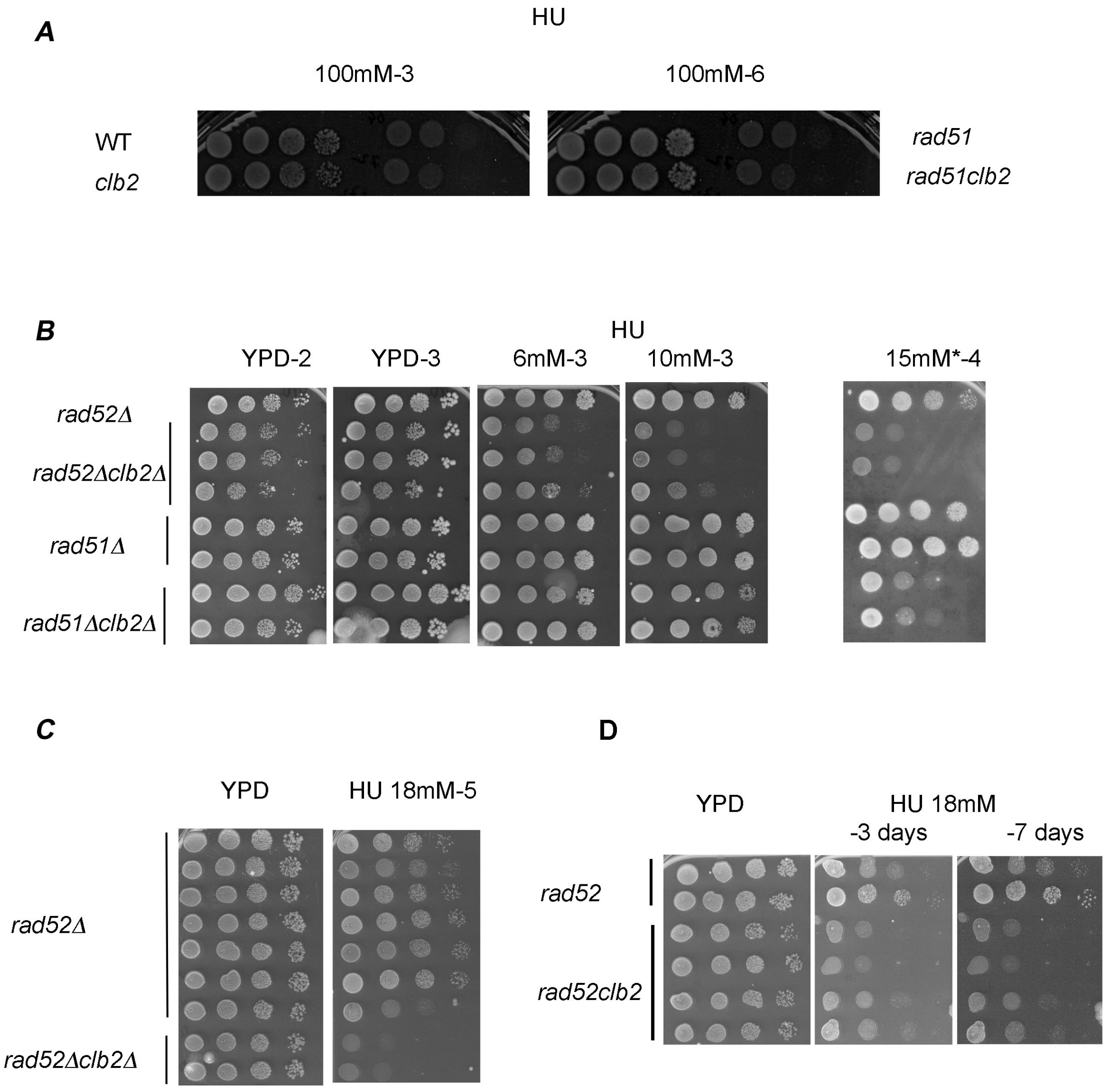

Figure S3. Additional *rad51,rad51clb2, rad52* and *rad52clb2* mutants and unstiches images of (A) Figure 4A. (B) Figure 4C. A variability of about 10-fold is observed at 6mM HU for two *rad52clb2* mutants out of the seven. Spots observed at the most concentrated cell densities for *rad51clb2* mutant correspond to dead cells (C) A variability of about 10-fold at 18mM is observed for one *rad52* mutant out of the seven (D) additional experiment and *rad52clb2* mutants.
Plates were incubated for -x days at 30°.

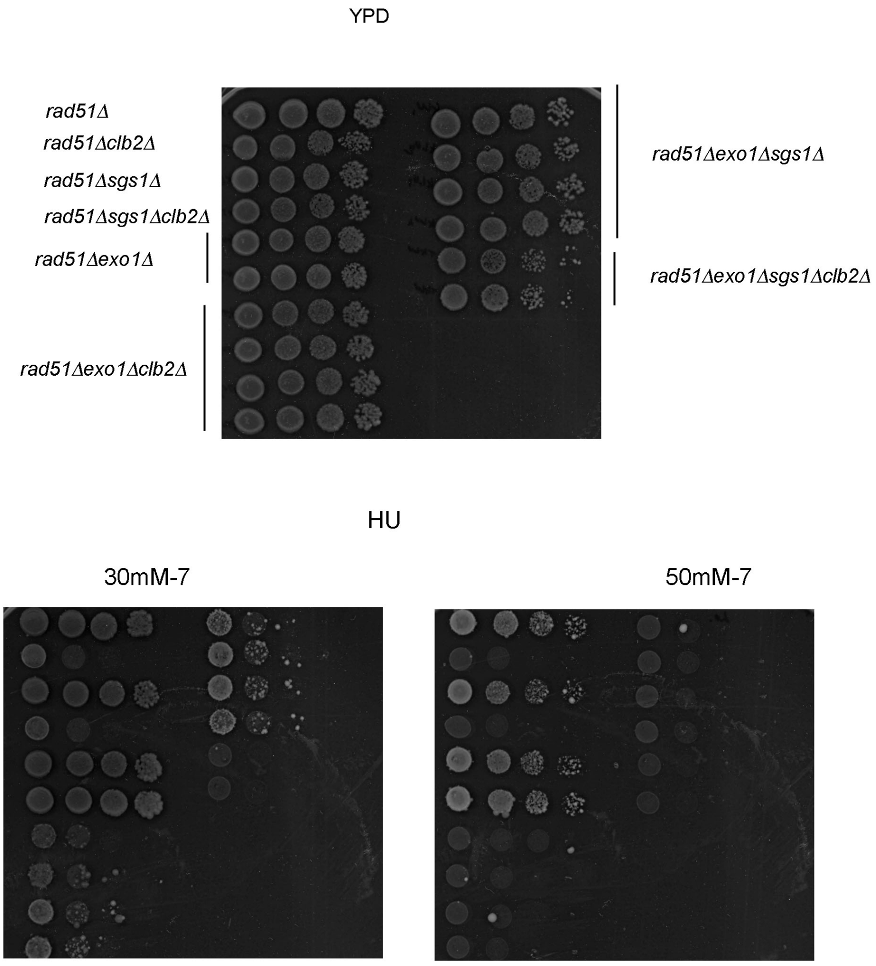

Figure S4. Unstiches image of Figure 4D and additional mutants

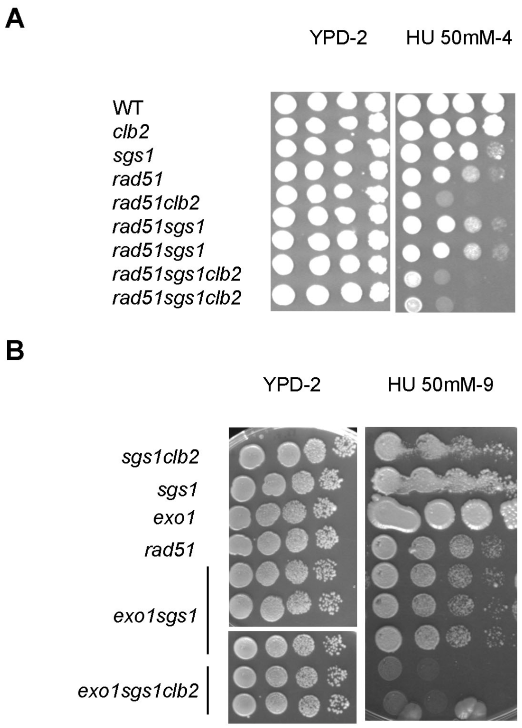

Figure S5. Additional experiments showing (A) genetic interaction between *RAD51, SGS1 and CLB2*. *rad51* mutant is slightly more sensitive than *sgs1* cells. (B) *rad51* cells display similar sensitivity to *exo1sgs1*and *sgs1clb2* mutants.

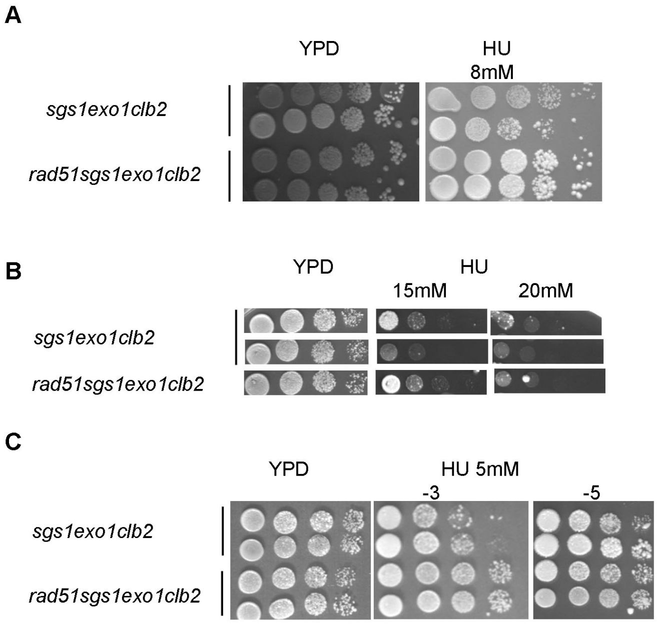

Figure S6. Additional experiments showing that *rad51sgs1exo1clb2* mutants have (A) (B) comparable sensitivity to one *sgs1exo1clb2* mutant though (C) growth of *sgs1exo1clb2* mutants is impeded. or that *rad51sgs1exo1clb2* mutants (A) (B) (C) is found to be about 10-50 times less sensitive at HU concentration ranging from8 to 15mM HU compared the other sgs1exo1clb2 mutant.

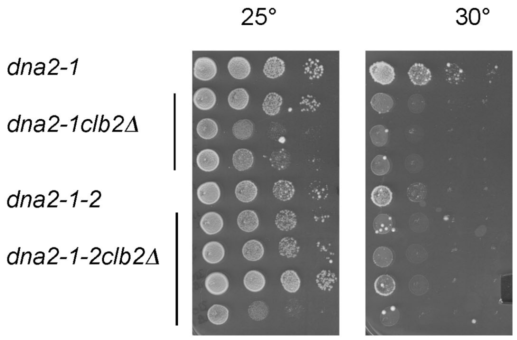

Figure S7. Additional experiment showing that *CLB2* deletion increases thermo-sensitivity of *dna2-1* mutants.Though variability is observed among *dna2-1clb2* mutants all *dna2-1clb2* mutants display an increased sensitivity of about 10- to 100-fold or more compared to *dna2-1* mutants.

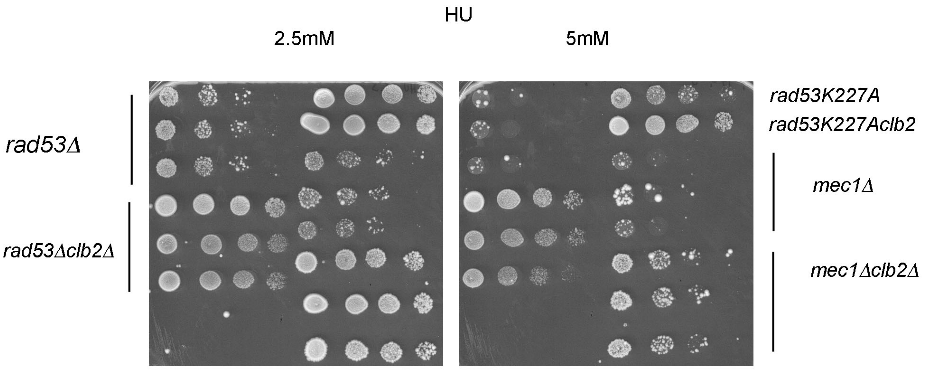

Figure S8. Unstiches image of Figure 6A and additional *mec1, mec1clb2, rad53* and *rad53clb2* mutants.

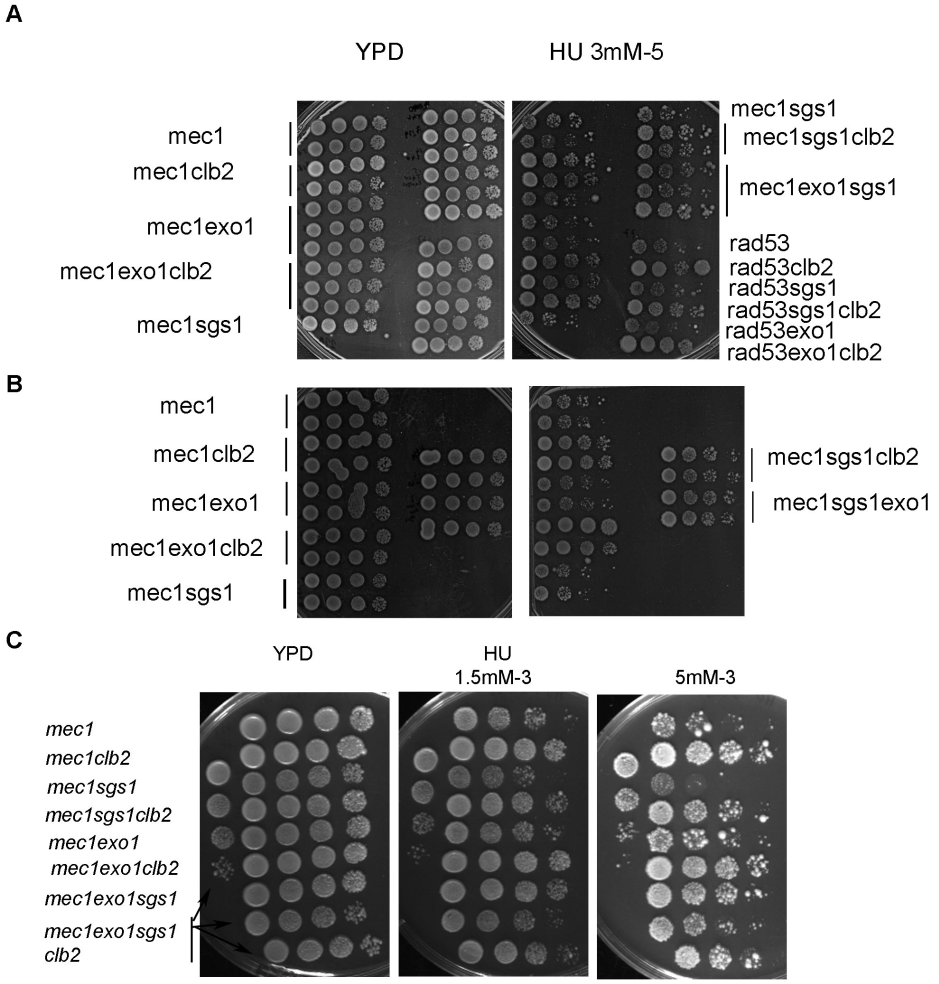

Figure S9. (A). Additional mutants and experiments in the *mec1* background. As shown in Figure S8, the three mec1 mutant obtained after transformation behave similarly. One *mec1* spores (obtained by dissection of diploid) was tested. that display small colony size in response to HU and a less than 10-fold difference in sensitivity to HU compared to corresponding. mec1 transformant.
(B) In another experiment, an additional *mec1* spore was tested and display similar sensitivity and colony size to the mec1 mutants obtained by transformation.
(C). Additional *mec1sgs1exo1clb2* mutants.

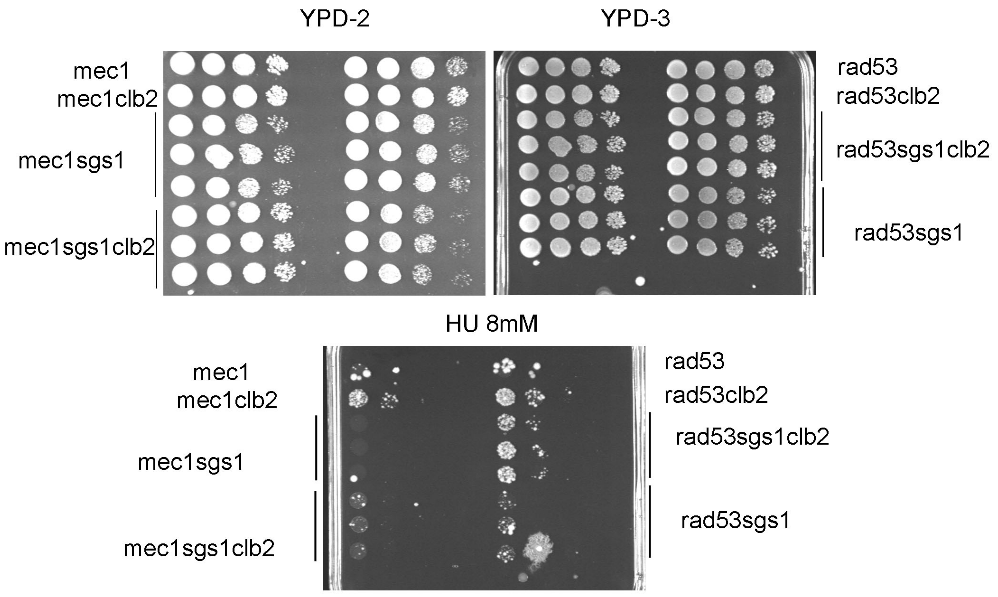

Figure S10. Additional *mec1sgs1, mec1sgs1clb2, rad53sgs1* and *rad53sgs1clb2* mutants. Growth of *rad53sgs1* mutants is impeded on YPD.

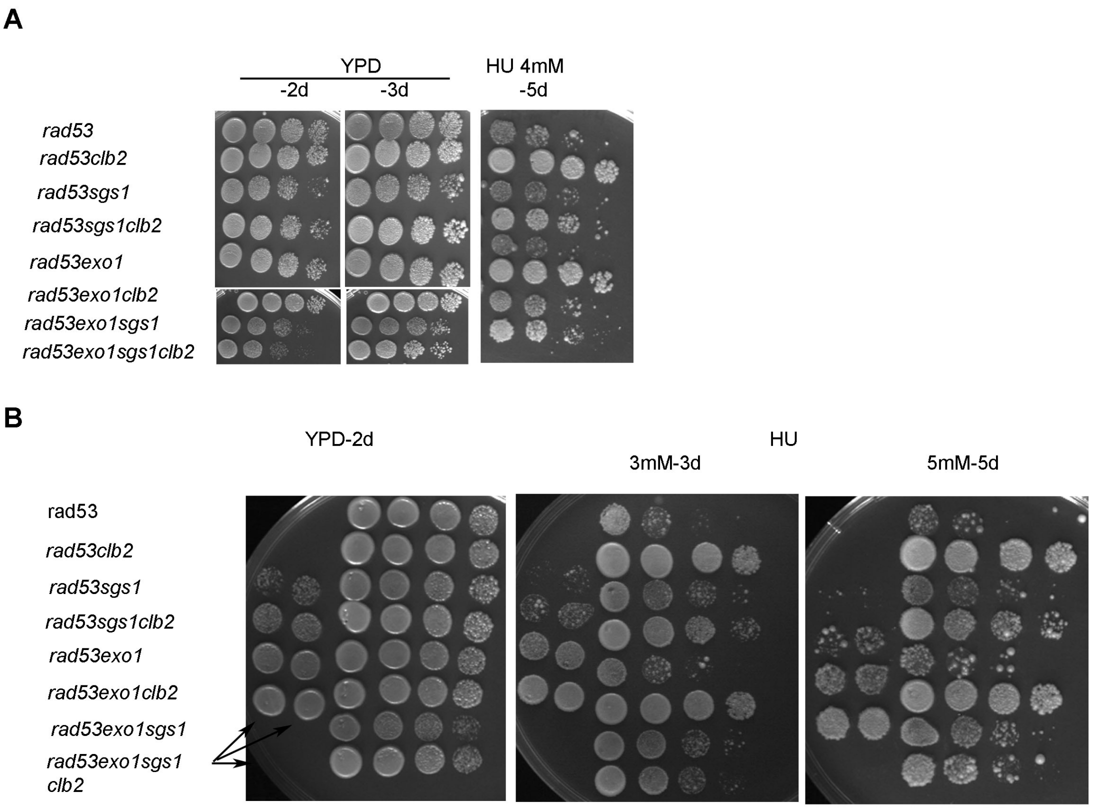

Figure S11. (A) Additional experiment showing the interaction of *CLB2, SGS1* and *EXO1* in *rad53* mutant. (B) Additional *rad53sgs1exo1clb2* mutants. -2d: 2 days of incubation at 30°.

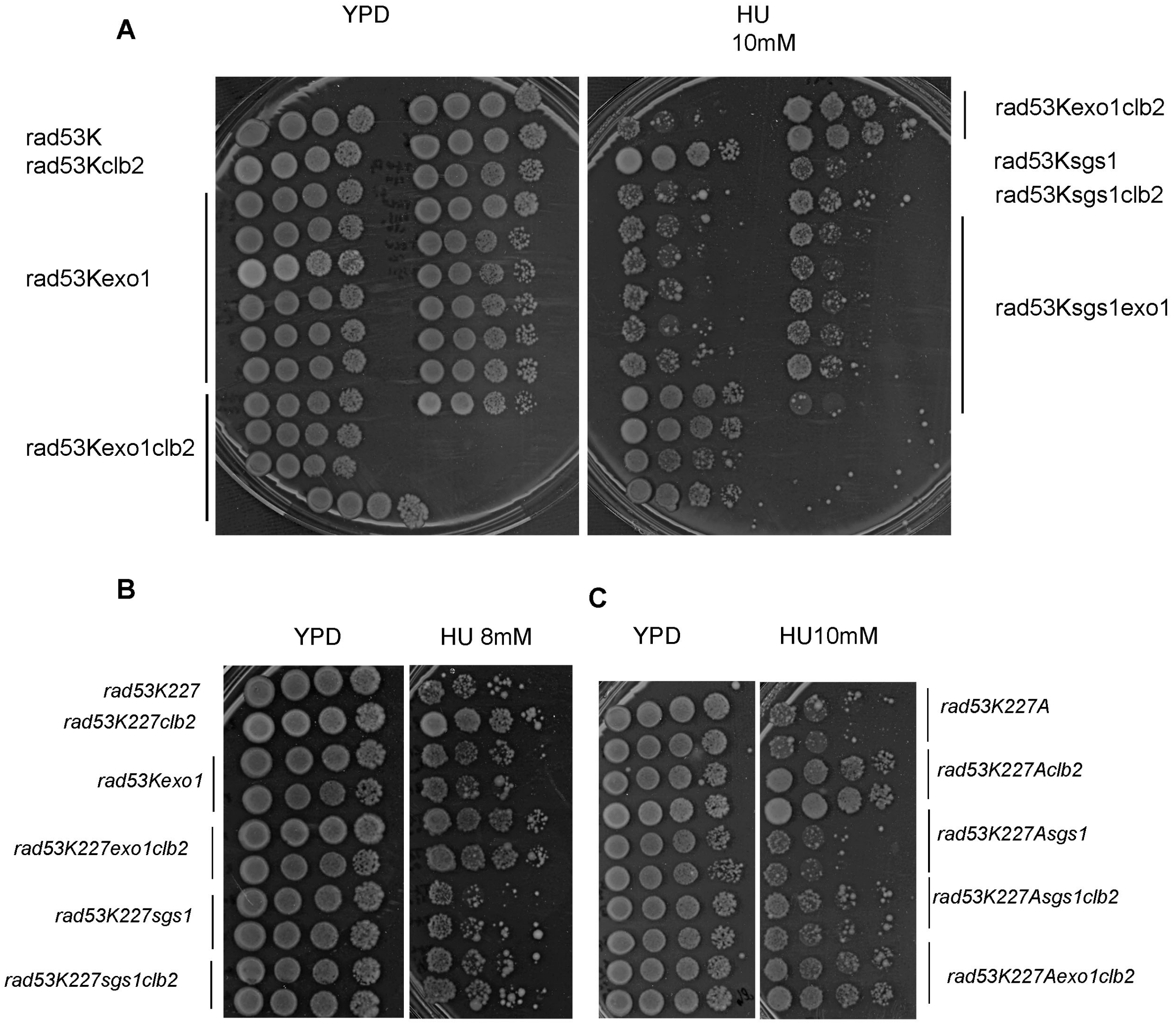

Figure S12. Genetic interaction between *CLB2*, *SGS1* and *EXO1* in *rad53K22A* mutant background. (A) Additional mutants and experiments. *rad53K227Aexo1clb2* mutants are 100- (5/6) to 10- times (1/6) less sensitive compared to *rad53K227A*. *rad53k227Aexo1sgs1* mutant display similar sensitivity to rad53K227A (five mutants out of six). One displays stronger sensitivity.(B,C) Additional experiments.
